# Electrochemical Biosensor Based on Hairy Core–Shell Particles: Effect of Core Conductivity

**DOI:** 10.1002/smsc.202500550

**Published:** 2026-04-07

**Authors:** Pavel Milkin, Anila Antony, Hongtao Cai, Ceyda Topal, Antonia Debevc, Anne Linhardt, Alla Synytska, Leonid Ionov

**Affiliations:** ^1^ Faculty of Engineering Sciences University of Bayreuth Bayreuth Germany; ^2^ Functional Polymer Interfaces Group Bayerisches Polymerinstitut (BPI) University of Bayreuth Bayreuth Germany

**Keywords:** bioelectrocatalysis, electrochemical biosensor, hairy core–shell particles, laccase, polymer brush

## Abstract

In this study, the effect of the core material in hairy core–shell carriers with grafted poly(2‐(dimethylamino)ethyl methacrylate) (PDMAEMA) polymer brushes containing immobilized laccase from *Trametes versicolor* (*TvL*) is investigated. Conductive silver, silver‐Janus, carbon nanotubes, carbon black, and insulating silica particles were chosen as core materials. These carriers are easy to handle and store, enabling reproducible sensor fabrication with a well‐defined and high number of immobilized enzymes. Hydroquinone (HQ) detection in an aqueous system was chosen as a model for biosensor characterization. All systems demonstrated high catalytic efficiency, significantly suppressing the oxidation of HQ at the electrode surface and thereby providing selectivity. The sensitivity of the prepared sensors did not differ significantly among the carriers. However, the limit of detection was highly dependent on the overall conductivity of the carrier and its active surface area. Our main observation is that using highly conductive, high‐surface‐area carriers does not necessarily enhance sensor performance and can in fact worsen the detection limit due to the dominance of capacitive currents over faradaic ones.

## Graphical abstract

Poly(2‐(dimethylamino)ethyl methacrylate)‐grafted hairy core–shell carriers with immobilized laccase were prepared on cores ranging from insulating SiO_2_ to highly conductive Ag, CNT, and CB. All systems enabled selective hydroquinone detection, but their LOD varied strongly. Excessive conductivity and surface area increased capacitive currents, reducing signal clarity. Thus, more conductive carriers do not necessarily yield better biosensor performance.

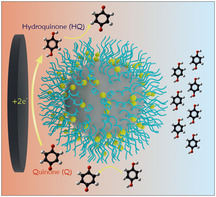

## 1 Introduction

Enzymatic electrochemical sensors offer a number of advantages such as high selectivity (certain enzymes catalyze certain redox reactions) and increased sensitivity due to acceleration of reaction rates [1, 2]. One of the most well‐known examples of enzyme‐based biosensors is glucose sensor, which plays a critical role in diabetes management. Glucose oxidase (GOx) or glucose dehydrogenase enzymes are generally used in these sensors and are widely preferred for blood sugar monitoring [3, 4]. Products such as *FreeStyle Libre* by *Abbott*, *Dexcom G7* by *Dexcom*, *Accu‐Chek Guide by Roche*, and *Contour Next* by *Ascensia* are prominent examples in this field [5]. In addition to glucose, various enzymes are used to measure many other metabolites. For example, lactate oxidase is used to measure lactate [6], cholesterol oxidase and esterase for cholesterol [7], and uricase for uric acid [8]. Furthermore, pollutants such as phenolic compounds can be detected using enzymes like laccase from *Trametes versicolor* (*Tv*L) or Tyrosinase, making them valuable tools in environmental monitoring applications [9, 10, 11]. These sensors have significant applications not only in the medical field but also across various domains, including healthcare, food safety, drug discovery, disease diagnosis, and environmental monitoring.

The performance of enzymatic biosensors is substantially dependent both on the rate of the catalyzed reaction, which depends on the amount of enzyme and their conformation and the rate of electron transfer, which depends on the transport rate, conductivity of electrode, and the surface area of electrode. Different methods of enzyme immobilization have been developed including adsorption, covalent binding, entrapment, cross‐linking, and affinity‐based methods [12, 13, 14]. It is expected that after immobilization, the structure and biological function of the enzymes should be preserved, and they should remain active. However, chemical binding often leads to conformational changes in enzymes, resulting in their partial denaturation or even degradation [15, 16]. Therefore, physical adsorption or electrostatic interaction is used as an alternative method to achieve high activity of immobilized enzymes. In particular, in our previous work, we demonstrated for the first time the achievement of high sensitivity and low detection limit by using an electrochemical sensor based on solid SiO_2_ carriers with a grafted poly(2‐(dimethylamino)ethyl methacrylate) (PDMAEMA) brush. In addition to a high sensitivity and very low detection limit, this method offers a number of other advantages, such as a quantifiable amount and activity of the immobilized enzyme, which increases the activity of the immobilized enzyme compared to that of a free one [17, 18, 19].

It is generally believed that the conductivity and surface area of the electrode must boost the performance of enzymatic biosensors because the measured signal is current, which scales with conductivity and surface area [20]. To elucidate if this assumption is fully correct, we investigate in this paper the effect of the particle core of core–shell hairy particles with immobilized enzyme on the performance of biosensors based on them. In addition to insulating silica particles, conductive carbon black (CB), carbon nanotubes (CNTs), silver (Ag), and silver‐Janus (Ag‐Janus) particles were also used. For completeness, the data for SiO_2_ carriers were taken from our previous work and are presented here as well [19]. All carriers were able to demonstrate catalytical oxidation of hydroquinone (HQ) in an aqueous solution, which was used as a model system. The choice of carrier did not significantly influence the sensitivity of the prepared sensors. However, due to differences in the surface properties of the carriers, highly conductive electrodes demonstrated the lowest limit of detection (LOD). The detailed mechanism behind this difference is discussed in this article.

## 2 Experimental

### 2.1 Chemicals

Tetraethyl orthosilicate (TEOS) (Sigma, 99%), ammonia solution (NH_4_OH, Sigma, 28–30% solution), absolute ethanol (EtOH, Sigma, 99.9%), copper(II) bromide (CuBr_2_, Sigma, 99.999%), (3‐aminopropyl)triethoxysilane (APTES, Sigma, 99%), α‐bromoisobutyryl bromide (Sigma, 98%), propionyl bromide (Sigma, 97%), tris(2‐pyridylmethyl)amine (TPMA, Sigma, 98%), tin(II) 2‐ethylhexanoate (Sigma, 95%), ethyl α‐bromoisobutyrate (EBiB, Sigma, 98%), Laccase from Trametes versicolor (Sigma, ≥0.5 U/mg), 2,2′‐azino‐bis(3‐ethylbenzthiazoline‐6‐sulphonic acid) diammonium salt (ABTS, Sigma, ≥98%), N, N‐Dimethylformamide (DMF, anhydrous, Sigma, 99.8%), dichloromethane (DCM, Extra Dry, Thermoscientific, 99.8%), Triethylamine (Et_3_N, Sigma, ≥99.5%), HQ (Sigma, 99%), potassium hexacyanoferrate (III) (K_3_[Fe(CN)_6_], Sigma, 99.98%), potassium chloride (KCl, Sigma, 99.0–100.5%), sodium acetate (CH_3_COONa, Sigma, ≥99%), acetic acid (CH_3_COOH, Sigma, ≥99.7%), sodium azide (NaN_3_, Sigma, ≥99.5%), Tris buffer (pH 8.5) (Carl Roth), dopamine hydrochloride (Sigma, > 97.5%), silver nitrate (Sigma, ≥99.0%), 2‐mercaptoethanol (Sigma, ≥99.0%), 11‐mercaptoundecyl 2‐bromo‐2‐methylpropanoate (Br‐initiator for Ag‐particles, TCI, >95.0%), polyvinylpyrrolidone (PVP, Sigma, Mw 40 000), hydrogen tetrachloroaurate(III) trihydrate (HAuCl_4_·3H_2_O, Acros, 99%), L‐ascorbic acid (Sigma, 99%), acetonitrile (Sigma, HPLC grade ≥99.9%), sodium borohydride (NaBH_4_, Sigma, 99%), and DCM (Acros, 99.99%) were used as received. 2‐(Dimethylamino)ethylmethacrylate (DMAEMA, Sigma, 98%) was passed through basic, neutral, and acidic aluminium oxide columns for 20 min to remove the inhibitor prior to polymerization. Multiwall CNTs (MWCNT, NC7000TM, 90% purity, average diameter = 9.5 nm, length = 1.5 µm), supplied by Nanocyl, Sambreville, Belgium, and TIMCAL SUPER C65 conductive CB, supplied by Nanografi Nanotechnology, were pretreated with absolute ethanol at room temperature for 2 h.

### 2.2 Synthesis of Hairy Core–shell Particles

#### 2.2.1 SiO_2_

The synthesis for SiO_2_ hairy core–shell particles is described in our previous studies [17, 18]. 200 nm SiO_2_ particles were synthesized using the Stöber method. In the first step, a seed solution was prepared by mixing 50 mL of ethanol and 3 mL of NH_4_OH (28–30%) in a clean 500 mL snap‐top bottle at 500 rpm for 3–4 min. After stirring at room temperature for 12 h, 50 mL of the resulting mother solution (100 nm SiO_2_ particles) was transferred to a new vial, and 350 mL of ethanol and 24 mL of NH_4_OH (28–30%) were added. After stirring for 3‐4 mins, 12 mL of TEOS was added dropwise. After stirring at room temperature for 12 h, the resulting 200 nm SiO_2_ particles were separated by centrifugation at 12 000 rpm for 10 min, washed five times with ethanol, and dried under vacuum at 60°C overnight.

The particle surface modification is also carried out in two steps. First, APTES modification was performed, and then, the bromine‐containing functional groups are modified. First, SiO_2_ particles (2 g) were placed in a 250 mL round‐bottom flask with a stirring bar, and 95 mL of ethanol and 5 mL of APTES were added. After stirring at room temperature for 12 h, the particles were separated by centrifugation at 12 000 rpm for 10 min, washed with ethanol 5 times, and vacuum dried at 60°C overnight. Second, 1 g of APTES‐modified SiO_2_ particles was added to a 100 mL round‐bottom flask with a stirring bar, 50 mL of DCM, stirred for 3 min. Then, 0.5 mL of α‐bromoisobutyryl bromide (BrIn) and 0.36 mL of propionyl bromide were added, and the mixture was stirred for 3 min. Following this, 2 mL of triethylamine was added, and the resulting solution was stirred at 700 rpm at room temperature. After 2 h, the particles were collected by centrifugation at 12 000 rpm for 10 min, washed twice with DCM and three times with ethanol, and dried overnight at 60°C in a vacuum.

500 mg of BrIn premodified silica particles were placed in a test tube with a septum and stir bar, and DMAEMA (5.9 mmol), anhydrous DMF (38.7 mmol, 3 mL), 30 μL CuBr_2_ (0.1 M in DMF, 3 × 10^−3^ mmol), TPMA (22.4 mmol), and EBIB (1 × 10^−3^ mmol) were added while stirring. The dispersion was purged with argon in an ultrasonic bath. After 10 min, the tin 2‐ethylhexanoate (Sn(II), 0.31 mmol) was added as a reducing agent. The reaction was carried out in an oil bath at 70°C and 800 rpm for 30 min. Polymer‐modified particles were collected by centrifugation, washed several times in DMF and absolute ethanol, and dried in a vacuum under reduced pressure at 25°C.

#### 2.2.2 Silver

Silver nanoparticles were synthesized and modified with PDMAEMA according to the procedure described in our previous work [21]. Typically, silver nanoparticles were synthesized in batches using a seeded‐growth method with gold nanoparticles serving as seeds. Au seeds were prepared by reducing HAuCl_4_ with NaBH_4_ in the presence of PVP, followed by ageing for 6 h to decompose excess reductant. For the silver particle growth, ascorbic acid was used as a mild reductant in a PVP‐stabilized aqueous system containing acetonitrile. AgNO_3_ was then added, followed by a small amount of the seed solution. After 2 h of reaction at 25°C, the resulting particles were collected by centrifugation, washed with ethanol, and stored in ethanol at 4°C. Silver nanoparticles (200 mg) were dispersed in 100 mL of ethanol, and 300 μL of Br‐initiator (11‐mercaptoundecyl 2‐bromo‐2‐methylpropanoate) was added for ligand exchange. After stirring for 12 h, the Ag@Br‐initiator particles were collected by centrifugation, washed with ethanol, and redispersed in 10 ml DMF for storage at 4°C. Ag@Br‐initiator particles (50 mg) were mixed with EBIB (0.2 × 10^−3^ mmol), 6 μL CuBr_2_ (0.1 M solution in DMF, 0.6 × 10^−3^ mmol), TPMA (4.5 mmol), anhydrous DMF (38.7 mmol, 3 mL), and DMAEMA (7.1 mmol). The solution was degassed under argon, sonicated (80 W, 10 min, ice bath), then initiated by injecting Sn(II) 2‐ethylhexanoate (61.8 × 10^−3^ mmol) in 2.6 mmol DMF. The polymerization proceeded at 70°C for 60 min. Products were purified by centrifugation, washed with DMF and ethanol, and redispersed in ethanol.

#### 2.2.3 Silver‐Janus

Silver‐Janus particles were prepared using the Pickering emulsion approach described in our previous work [22]. Spherical Ag particles (1 g, *Ø* ≈ 170 nm) were dispersed in 100 mL of chloroform and ultrasonicated for 1 h in a covered round‐bottom flask. After heating to 50°C, paraffin wax (10 g) was added and further dispersed until fully dissolved. Chloroform was then removed via rotary evaporation (50–60°C, 450–80 mbar). The resulting Ag–wax mixture was stirred at 85°C and up to 800 rpm. Preheated deionized (DI) water (100 mL, 90°C) was added, followed by high‐speed stirring (1100–1300 rpm) at 85°C for 1 h to form a wax‐in‐water emulsion. The emulsion was quenched in liquid nitrogen, thawed, filtered, and thoroughly washed with DI water. The recovered colloidosomes were dried under vacuum at 25°C overnight. The dried colloidosomes were redispersed in 100 mL of ethanol, and 300 μL of 11‐mercaptoundecyl 2‐bromo‐2‐methylpropanoate was added. The reaction proceeded for ≥48 h at < 5°C and 400 rpm. Modified colloidosomes were filtered, washed with 1 L ethanol, and vacuum‐dried at 25°C overnight. The initiator‐modified colloidosomes were washed five times with hot hexane (60°C), followed by two times with DCM and three times with ethanol. After removing the waxy phase, the Janus particles were dispersed in 5 mL DMF for polymer grafting. For ATRP, 50 mg of Janus Ag particles were mixed with DMAEMA (5.9 mmol), anhydrous DMF (38.7 mmol, 3 mL), EBIB (0.2 × 10^−3^ mmol), 6 μL CuBr_2_ (0.1 M in DMF, 0.6 × 10^−3^ mmol), and TPMA (4.5 mmol). The mixture was purged with argon, sonicated (80 W, 10 min, ice bath), and initiated with Sn(II) 2‐ethylhexanoate (61.8 × 10^−3^ mmol) in 2.6 mmol DMF. Polymerization was conducted at 70°C for 60 min. The final particles were centrifuged (4000 rpm, 20 min), washed with DMF and ethanol, and redispersed in 10 mL of ethanol.

#### 2.2.4 Carbon Black and Carbon Nanotubes

The first premodification step of CB or CNTs is surface functionalization using a polydopamine (PDA) layer, inspired by muscle chemistry. 1 g of prewetted native particles was dispersed in 250 mL of 50 mM Tris buffer with pH 8.5 using ultrasonication (Fisherbrand ultrasonicator probe (120 W) with 100% amplitude was used throughout the experiment for CNT and CB) in a round‐bottom flask with stirrer bar and septum. 400 mg dopamine hydrochloride dissolved in Milli‐Q water was added dropwise under magnetic stirring. The reaction was continued for 24 h at room temperature and 800 rpm. Then, PDA‐modified particles were washed with absolute ethanol and milliQ‐water several times and dried in a vacuum oven under reduced pressure at 60°C.

Afterwards, these particles were functionalized with the bromine initiator, α‐bromoisobutyryl bromide (BrIn). 250 mg PDA‐modified CB, or CNT was dispersed in 50 mL of DCM. While magnetically stirring the solution, 1 mL BrIn was added, followed by the addition of 2 mL triethylamine. The reaction was continued overnight at room temperature and 800 rpm. The BrIn‐modified particles were washed three times with DCM and six times with absolute ethanol and dried in a vacuum oven under reduced pressure at 60°C.

For grafting polymer brush using ATRP, 20 mg of BrIn‐modified CB or CNTs were placed in a 25 mL round‐bottom flask with a magnetic stirrer bar and dispersed in DMF. DMAEMA (5.9 mmol), anhydrous DMF (51.7 mmol, 4 mL), EBiB (1 × 10^−3^ mmol), TPMA (22.4 mmol), and 30 µL CuBr_2_ (0.1 M solution in DMF, 3 × 10^−3^ mmol) were added, followed by high power ultrasonication for 30 s. Then, the round‐bottom flask was sealed with a septum, and the mixture was purged with argon gas for 10 minutes while being sonicated in an ultrasound ice bath. This was followed by the injection of a reducing agent, tin 2‐ethylhexanoate (Sn(II), 0.31 mmol), dissolved in 12.9 mmol (1 mL) of anhydrous DMF. The polymerization was carried out by immersing the system in a preheated oil bath at 60°C with 700 rpm for 30 min. Polymer‐modified particles were collected by centrifugation, washed several times in DMF and absolute ethanol, and dried in a vacuum under reduced pressure at 25°C.

### 2.3 Immobilization of Laccase and Activity Measurements

Polymer‐modified particles (SiO_2_, Ag, Ag Janus; 10 mg and CB, CNT; 2 mg) were washed thrice with 10 mM sodium acetate pH 4 buffer and dispersed in 500 µL of buffer. 500 µL of laccase enzyme solution with desired activity in sodium acetate buffer was added and immobilized at room temperature for 1 h under gentle shaking. This was followed by collecting the supernatant and washing the particles with sodium acetate buffer using centrifugation (8000 rpm, 5 min, RT) until no enzyme activity was measured in the supernatant. Finally, the particles were dispersed in 1 mL buffer.

A Tristar5 multifunctional microplate reader (Berthold Technologies) was used to measure the activity of enzymes at 25°C. The laccase activity was determined using the ABTS assay at 420 nm. Laccase oxidizes the substrate ABTS to the cation radical ABTS^+^ with a colour change from colourless to green, which was measured by UV/Vis spectroscopy at 420 nm.

The activity of the enzymes was calculated by the absorbance change (ΔE) per time interval (Δt) divided by the extinction coefficient of the substrate (ε_ABTS_, 36,000 M^−1^ cm^−1^) times the path length (d) (Equation (1)).

(1) $$\text{Activity}=\;\frac{\frac{\Delta E}{\Delta t}}{\varepsilon \cdot d}$$

The enzyme activity on particle or polymer was calculated using Equation (2).

(2) $$\text{Activity}\left(\frac{U}{mg\;\text{particle or polymer}}\right)=\frac{\text{Activity of immobilized enzyme}}{\text{mass of particle or polymer}}$$

### 2.4 Modification of Working Electrode and Electrochemical Assays

The commercial screen‐printed electrodes (SPEs) were modified by drop‐casting of a particle suspension in a 0.1 M pH = 4 acetate buffer onto the working electrode (3 mm diameter) followed by drying in air (Table 1). After drying, the modified electrodes were left in the acetate buffer overnight at 4°C. Prior to the measurements, the electrodes were cleaned by cycling the electrodes 50 times in the range of −0.8 to 0.8 V at 100 mV/s in pure acetate buffer to ensure that there are no contaminating signals. The electrical impedance spectroscopy (EIS) measurements were performed at the formal potential of the redox couple with an amplitude of 10 mV in the frequency range of 0.1–10^6^ Hz. All electrochemical measurements were performed using Gamry 1010E potentiostat.

**TABLE 1:** Parameters of carriers modified by PDMAEMA brushes with immobilized laccase used for SPE modification.

Carrier	Volume of suspension on electrode, µL	Initial concentration of enzymes, U/mL	Initial concentration of enzymes, mg/mL	Carriers activity, U/mL	Carriers concentration, mg/mL	Activity U/mg of particles	Absolute activity on electrode, U
CNT	20	37	140	15.2	2	7.6	0.3
CB	20	10.7	40	7.3	2	3.6	0.15
Ag	10	27.2	108	13.8	10	1.38	0.138
Ag Janus	10	22.0	81	9.7	10	1.0	0.10
SiO_2_	10	16	50	11.3	10	1.13	0.113

For further investigation of charge transport, the heterogeneous rate constant k_0_ was evaluated using the following Equation (3):

(3) $$k_{0}=\frac{RT}{n^{2}F^{2}AR_{ct}C}$$

where *n* is the number of electrons transferred during redox process, *A* is the active surface area (cm^2^), *R*_*ct*_ is the charge transfer resistance obtained from EIS data, *C* is the concentration of electroactive species (mol cm^−3^), *R* is the ideal gas constant (8.314 J mol^−1^ K^−1^), *T* is the temperature, and *F* is the Faraday constant (96485 C·mol^−1^). The active surface area *A* was calculated using Randles–Sevcik Equation (4)

(4) $$i_{p}=0.446nFAC{\left(\frac{nFvD}{RT}\right)}^{\frac{1}{2}}$$

where *i*_*p*_ is the is reduction peak, *A* is the electroactive surface area of electrode, *C* is the bulk analyte concentration, *v* is the scan rate, *F* is the Faraday constant, *D* is the diffusion coefficient, and *T* is the temperature.

### 2.5 Other Analytical Methods

#### 2.5.1 Thermogravimetry

The thermogravimetry (TGA) was performed using a TG 209 F3 Tarsus (Netzsch) at a heating rate of 10 K·min^−1^ in a nitrogen atmosphere. Considering SiO_2_, Ag, and Ag Janus particles as spherical, the polymer brush thickness *t* was calculated according to Equation (5):

(5) $$t=\;\frac{R*\;\rho_{\text{particle}}*\varphi_{\text{Polymer}}}{3\cdot \rho_{\text{polymer}}*(1-\varphi_{\text{Polymer}})}$$

where *R* is the particles radius, *ρ*_particles_ is the density of particles (SiO_2_: 2.4 g·cm^−3^, Ag: 10.5 g·cm^−3^, CB: 1.6g·cm^−3^), *φ* is the PDMAEMA mass fraction, and $\rho_{\text{polymer}}$ is the PDMAEMA density (1.318 g·cm^−3^).

In contrast, the CNTs are considered as cylinders, and Equation (6) is used to calculate the polymer brush thickness.

(6) $$t=\frac{d\cdot \rho_{CNT}*\varphi_{\text{Polymer}}}{4\rho_{\text{polymer}}*\left(1-\varphi_{\text{Polymer}}\right)}$$

where *d* is the diameter of CNT (9.5 nm), and *ρ*_CNT_ is the density of CNT (1.66 g·cm^−3^)

#### 2.5.2 Gel Permeation Chromatography (GPC)

The number average molecular mass *M*_*n*_, the weight average molecular mass *M*_*w*_, and polydispersity index (PDI) (PDI *= M*_*w*_ */M*_*n*_), were determined by GPC (Agilent 1260 Infinity). The measurements were carried out at 60°C. The eluent for GPC measurements was DMF of high‐performance liquid chromatography grade with addition of 0.01 M LiBr, and the standards used for calibration were monodisperse poly(methyl methacrylate). The molecular weight of the free polymer obtained after centrifugation was determined at a flow rate of 0.5 mL·min^−1^. Based on the measured *M*_*n*_, the grafting density (GD) of the polymer brush was calculated according to Equation (7)

(7) $$GD=\;\frac{t*\;\rho_{\text{polymer}}*N_{A}}{M_{n}}$$

where *t* is the polymer brush layer thickness calculated from TGA, *ρ*_polymer_ is the density of the polymer, *N*_*A*_ is the Avogadro number, and *M*_*n*_ is the average molecular weight of the polymer determined by GPC.

## 3 Results and Discussion

### 3.1 Synthesis of Hairy Core–shell Particles (Enzyme Carriers) and Enzyme Immobilization

**FIGURE 1:**
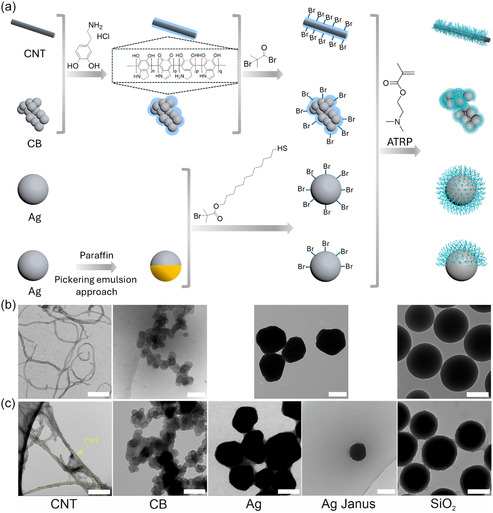
(a) Scheme of synthesis of various hairy core–shell particles (CNT, CB, Ag, and Ag‐Janus) as well as their TEM image of (b) bare particles and (c) with grafted PDMAEMA brush (scale bar 150 nm). The synthesis of SiO_2_ carriers was discussed in detail in our previous study [19].

To study the influence of polymer brush‐modified core materials with immobilized enzyme on the amperometric sensor performance, CNT, CB, Ag, and SiO_2_ have been used as carriers. Initially, the particles are premodified to attain a bromoinitiator surface functionalization for the successful ‘grafting‐from’ of PDMAEMA brushes via surface‐initiated atom transfer radical polymerization (ATRP) (Figure 1). The first premodification step of CNT and CB includes the modification with a thin layer of PDA through the self‐polymerization of dopamine hydrochloride in an alkaline medium [23]. Although the mechanism of dopamine self‐polymerization and the self‐assembled structure of the PDA are not fully understood [24], the high concentration of catechol and amine groups on the PDA surface makes it capable of anchoring an initiator (α‐bromoisobutyryl bromide, BrIn) for ATRP to the surface of the CNT and CB particles. The silver nanoparticles (Ag NPs) were synthesized as described in the synthesis section. To enable surface‐initiated polymerization, the Ag NPs were functionalized with a thiol‐based initiator via ligand exchange. Specifically, a thiol‐terminated ATRP initiator was used to replace the weakly adsorbed stabilizers on the Ag surface, exploiting the strong affinity between thiol groups and silver. This surface modification was performed under mild conditions in ethanol, ensuring a stable and dense initiator layer for subsequent polymer brush growth.

The morphology change after polymer modification is characterized using transmission electron microscopy (TEM ‐ Figure 1, SEM ‐ Figure S2). TEM images clearly show the polymer brushes grown from the nanoparticle surfaces, forming polymer bridges. Apart from electron microscopy, we performed TGA analysis to evaluate the polymer brush thickness on each kind of nanoparticle carrier (Figure S1). After polymer grafting, mass loss for CNT, CB, Ag, and Ag‐Janus carriers was around 47, 13, 10.7, and 10.1%, respectively. The polymer brush thickness was calculated according to Equations (5) and (6) as 2.7, 2.12, 23, and 13 nm, respectively (Table 2). Carbon particles (CB and CNTs) showed the lowest GD of the polymer, while silver particles and SiO_2_ showed the highest values. The most probable reason for this difference is the different chemistry used for the surface modification of nanoparticles. Apparently, PDA‐based approach (used for carbon particles) is less efficient and provides fewer functional groups than thiol‐ and silane‐based ones used for silver and SiO_2_ particles, respectively. Higher brush thickness on silver‐based particles is explained by a higher monomer‐to‐initiator ratio (Table 3). Thus, we have nonconductive SiO_2_ particles with relatively dense brush, conductive silver particles with dense, and thick brush and conductive CNT and CB particles with sparse and thin brush layer.

**TABLE 2:** Parameters of carriers modified by PDMAEMA brushes with immobilized laccase.

Carrier	Brush thickness (TGA), nm	**Grafting density, chains/nm** ^**2**^	Polymer molecular weight, kDa		Thickness of enzyme layer (TGA), nm	Activity		
			**M**_**n**_	**M**_**w**_		**U/mg** **of particles**	**U/mg** **of polymer**	**U/mg** **of** **enzyme**
CNT	2.7	0.13	17.1	34.5	0.50	7.6 ± 0.9	16.1 ± 2	53 ± 6
CB	2.12	0.08	13.7	20.6	1.96	3.6 ± 0.3	27.6 ± 3	29.6 ± 3
Ag	23	0.3	79	141	21.9	1.1 ± 0.1	9.8 ± 0.7	8.9 ± 2
Ag Janus	13	0.14	70	135	41.8	1.0 ± 0.3	34.9 ± 5	4.4 ± 1
SiO_2_	6.01	0.26	18.4	26.2	3.70	1.13 ± 0.1	13.6 ± 1	21.8 ± 2

**TABLE 3:** Polymerization conditions for different carrier systems.

Carrier	Mass of particles, mg	**DMAEMA,** **mmol**	EBiB, mmol	** CuBr**_**2,**_ **mmol**	TPMA, mmol	Sn(II), mmol	Reaction conditions
CNT	20	5.9	1 × 10^−3^	3 × 10^−3^	22.4	0.31	60°C, 30 min
CB	20	5.9	1 × 10^−3^	3 × 10^−3^	22.4	0.31	60°C, 30 min
Ag	50	7.1	0.2 × 10^−3^	0.6 × 10^−3^	4.5	61.8 × 10^−3^	70°C, 60 min
Ag Janus	50	5.9	0.2 × 10^−3^	0.6 × 10^−3^	4.5	61.8 × 10^−3^	70°C, 60 min
SiO_2_	500	5.9	1 × 10^−3^	3 × 10^−3^	22.4	0.31	70°C, 30 min

The electrokinetic potential changes with pH of all nanocarriers were analyzed using a zetasizer. As depicted in Figure S2a,b, the native particles are mostly negatively charged, and the PDMAEMA modifications lead to positive charge (∼40 mV) up to approximately pH 9. Upon polymer modification, the isoelectric point (IEP) of CNT and CB, at pH 3.9 and 3.8, shifts to pH 9.2 and 9.3. The IEP of Ag particles at pH 3 shifts to pH 9.4 for fully polymer‐modified Ag and to pH 9.0 for Ag‐Janus. As expected, the partial modification of Ag‐Janus particles leads to a lower IEP due to incomplete surface coverage by grafted polymer. The reference SiO_2_ particles show an IEP of 9.5. Summing up, the IEP of CB‐PDMAEMA and CNT‐PDMAEMA is slightly lower than that of Ag‐PDMEMA and SiO_2_‐PDMAEMA, indicating a probably lower GD and thus a greater influence of the core material on the properties of the resulting system. It is important to note that grafting of PDMAEMA to all particles was successful and resulted in the shift of IEP to allow noncovalent immobilization of enzyme. Indeed, laccase is negatively charged at pH 4. The high positive potential of polymer‐modified particles at pH 4, due to the protonation of the tertiary amine groups, helps the efficient adsorption of laccase via electrostatic interaction [19].

Enzyme immobilization and the ABTS assay were performed in 10 mM sodium acetate buffer at 25°C. Each carrier system was studied with immobilization of different initial enzyme activities, and the assay was performed to identify the total enzyme activity that was immobilized (Figure S3). The carrier systems showed the highest enzyme immobilization across various initially added enzyme activities. This indicates that the enzyme immobilization efficiency varies between systems, and they achieve a saturation limit. The carrier systems with the highest immobilization activity were chosen for further electrochemical experiments. The immobilized enzyme activity of selected carrier systems of CNT, CB, Ag, and Ag Janus was 15.2, 7.3, 13.8, and 9.7 U·mL^−1^, respectively (Table 1). The mass loss shown after the enzyme immobilization by TGA also confirms the successful immobilization of laccase on all particles (Figure S1). The most interesting values derived from TGA and activity measurement experiments are (i) the thickness of the enzyme layer calculated using TGA results, which characterizes the amount of immobilized enzyme (*N*_*E*_), the ratio between the thickness of the enzyme layer and the brush layer shows the efficiency of brush from point of attraction of enzyme; (ii) activity of the enzyme per its weight *A*_enzyme_; (iii) activity of the enzyme per mg of polymer, which is a product of amount of enzyme and activity of each enzyme molecule $A_{\mathrm{per}\;\mathrm{mg}\;\mathrm{of}\text{polymer}}∼N_{E}\cdot A_{\text{enzyme}}$.

**FIGURE 2:**
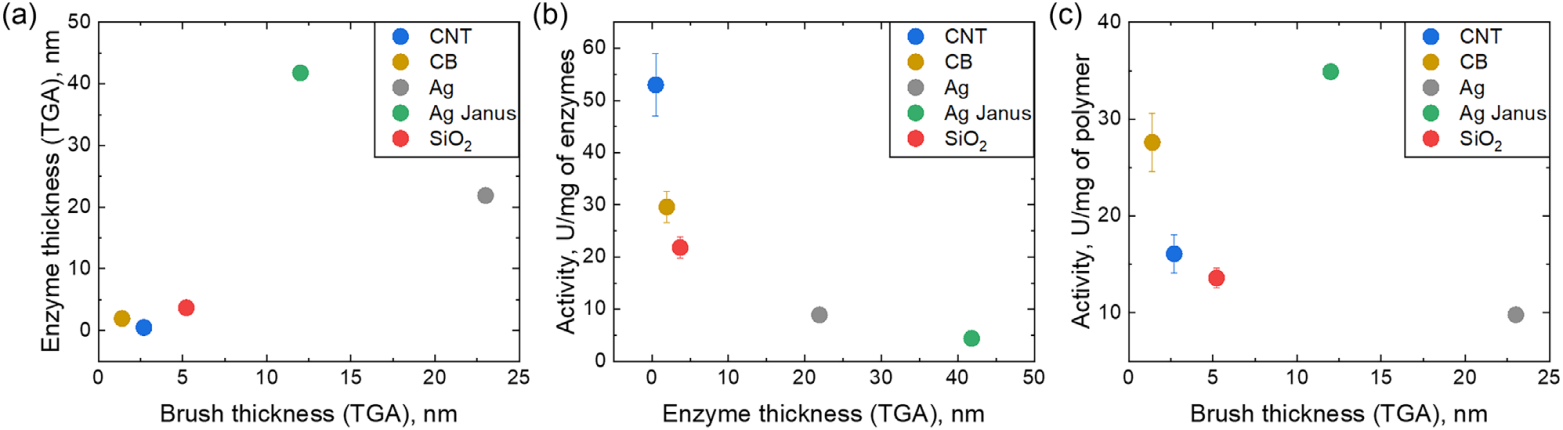
Dependencies of enzyme immobilization on various carriers: (a) enzyme thickness (TGA) vs brush thickness; (b) Activity of enzymes per mg of enzyme vs enzyme thickness (TGA); (c) activity of enzymes per mg of polymer vs brush thickness.

There is a general trend that the amount of immobilized enzyme increases with brush thickness. A thicker brush provides a larger available area for enzyme adsorption, especially considering that polymer brushes are swollen and can allow enzyme penetration into their interior (Figure 2a). However, at the largest brush thickness, the activity of immobilized enzyme decreases (Figure 2b,c). This could be attributed to limited enzyme penetration through a dense, thick brush layer [17, 18, 22], resulting in enzyme activity decrease per both mass of polymer and enzyme. Possible reasons include steric hindrance between closely packed enzyme molecules resulting in a nonoptimal enzyme conformation or competition for substrate binding.

Thus, all the carrier systems, CNT, CB, Ag, and Ag Janus modified with PDMAEMA brushes are capable of immobilization of laccase with enhanced enzyme activity, which are accurately measurable using ABTS assay and TGA analysis. All these immobilized systems are readily dispersible in sodium acetate buffer, making it easy to modify electrodes via drop casting. We could also calculate the amount of enzyme drop‐cast on each electrode, making measurements controlled from batch to batch.

### 3.2 Electrochemical Characterization of Electrodes

**FIGURE 3:**
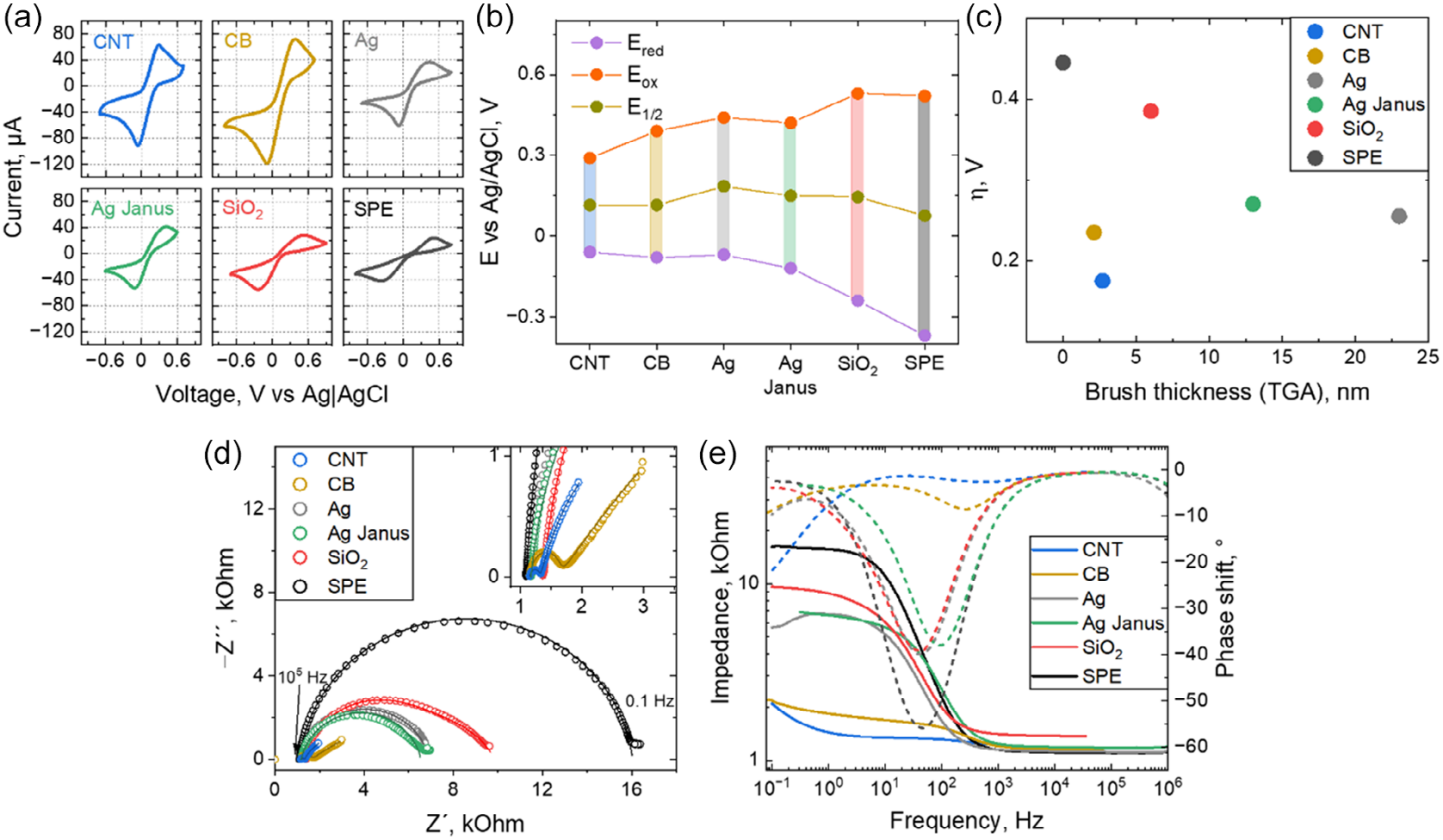
(a) CV of SPE and SPE‐modified electrodes, measured in solution containing 5 mM K_3_[Fe(CN)_6_] and 0.1 M KCl; scan rate 50 mV/s. (b) Reduction, oxidation, and formal potentials obtained from CV measurements (c) reduction overpotential obtained from CV vs. brush thickness of carriers; (d) Nyquist plot and (e) Bode plot of EIS results for SPE and SPE modified electrodes obtained in the same solution at formal potential offset with 10 mV amplitude.

The electrochemical properties of bare and modified SPEs were assessed using cyclic voltammetry with the commonly used [Fe(CN)_6_]^−3^/^−4^ redox couple, which served as a reference (Figure 3a,b). It is assumed that the transition between these two redox states is not catalyzed by laccase. As analyte, a 5 mM solution of K_3_[Fe(CN)_6_] in 0.1 M KCl was used – most of Fe ions in solution are in oxidized form – Fe(III), which is more stable than Fe(II). The bare SPE exhibited broad and weak anodic and cathodic peaks with a large peak‐to‐peak separation value (Δ*E* = 0.89 V), significantly higher than the theoretical Δ*E*_p_ = 59 mV (Δ*E*_p_ = 59/n mV at 25°C, where *n* is the number of electrons involved in redox process), indicating quasi‐reversibility of the process where reaction is not fast enough to maintain equilibrium during the scan and electrodes become polarized. Modification with nonconductive SiO_2_ – PDMAEMA particles slightly decreased the peak‐to‐peak separation, but not significantly (Δ*E* = 0.77 V). Moreover, the shape of the waves became less symmetrical with a more pronounced cathodic peak. We attribute the asymmetry of the peaks in both cases to the partial diffusion of Fe(II) complex to the bulk from electrode proximity due to slow electrode kinetics. The introduction of charged PDMAEMA brushes leads to even slower diffusion of the analyte. Conductive Ag, Ag Janus, and carbon carriers on the SPE lead to a decrease in peak‐to‐peak separation (up to Δ*E* = 0.35 V for the CNT carrier) and lower overpotential. We attribute this to the conductive nature of the carriers, as well as the possible adsorption of ferro/ferricyanide anions within the polycationic brushes, which facilitates the diffusion of the redox species to the electrode. This shift suggests a more reversible process with enhanced charge transfer. Despite the conductive nature of silver carriers, due to insulation by the much thicker brush, it still results in a relatively large Δ*E* value. In addition, the similar values of Δ*E* for the Ag and the Ag Janus carriers can be an indication of the Janus‐carriers agglomeration by their conductive side. Thus, the use of a conductive filler with a short brush, as in the case of carbon carriers, results in faster electron transfer. However, a long brush insulates the surface of conductive carriers, introducing additional kinetic limitations, as observed in the case of silver carriers.

EIS of bare and modified electrodes in the same solution was conducted to investigate charge transfer (Figures 3d and S6). Measurements were performed in the same solution at the formal potential of the redox couple with a 10 mV voltage amplitude. The results were fitted using a circuit model containing solution resistance (*R*_*s*_) in series with one or two parallel R‐CPE circuits (Tables 4 and S5). One R‐CPE circuit corresponds to the charge transfer resistance (*R*_ct1_) and double‐layer capacitance (CPE_1_). The second R_ct2_‐CPE_2_ circuit we attribute to the charge transfer resistance and double‐layer capacitance related to slower diffusion of polycationic PDMAEMA brushes.

**TABLE 4:** Electrochemical characterization of bare and particle‐modified SPE electrode in 5 mM K_3_[Fe(CN)_6_] + 0.1 M KCl solution.

	SPE	CNT	CB	Ag	Ag Janus	**SiO**_**2**_
ΔE_p_, V	0.89	0.35	0.47	0.51	0.54	0.77
R_ct_, kOhm	14.9	0.18	0.49	5.8	5.4	8.5
k_0_, 10^−3^·cm·s^−1^	0.22	3.9	1.0	0.23	0.20	0.15

The unmodified SPE electrode exhibited the highest charge transfer resistance (*R*_ct_) (Table 4). Introducing nonconductive SiO_2_‐based, brush‐decorated particles reduced the *R*_ct_ by half. This change is attributed to the polyelectrolyte nature of the polymer brush, which locally increases ionic strength and decreases the Debye length (*λ*_*D*_ *∼I* ^−1/2^, *I* – Ionic strength of electrolyte). Introducing silver carriers further decreased the *R*_ct_, as the conductive nature of the particles enhances charge transfer, despite the relatively thick brush slightly insulating the electrode surface. The lowest charge transfer resistance was observed for carbon particles. It correlates with the values of the heterogeneous rate constant k_0_, which was evaluated, using Equation (3). The active surface area *A* was calculated using the Randles–Sevcik Equation (4). As expected, the bare SPE electrode exhibits one of the lowest rate constants (Table 4). The addition of nonconductive SiO_2_ particles results in a slight decrease in the charge transfer rate constant. Ag and Ag Janus fillers did not result in a significant increase in k_0_ value. For carbon fillers, the k_0_ is ca. ten times higher, proving boosted charge mobility, which is important for the design of working electrode of the sensor. Since the average brush thickness of the CNT and CB carriers is lower, the formed double layer is thinner and occurs at a lower time scale (Figure 3e). However, due to the presence of an open surface, at longer time scale, the next drop in the phase shift is observed as a result of charging the internal surfaces of carbon materials, as it is typically observed for carbon‐based supercapacitors [25]. Thus, surface modification with insulating SiO_2_ particles and carriers grafted with a large PDMAEMA brush, as in the case of Ag and Ag‐Janus carriers, does not significantly alter the electrochemical behaviour of the bare carbon electrode. In contrast, modification with conductive CNT and CB carriers grafted with shorter polymer brushes results in a pronounced decrease in peak‐to‐peak separation and an increase in the heterogeneous rate constant, indicating faster electron transfer at the electrode interface. However, at large time scale, the charging of carbon materials is observed which may result in overlapping with low faradaic current.

### 3.3 Bioelectrocatalysis

**FIGURE 4:**
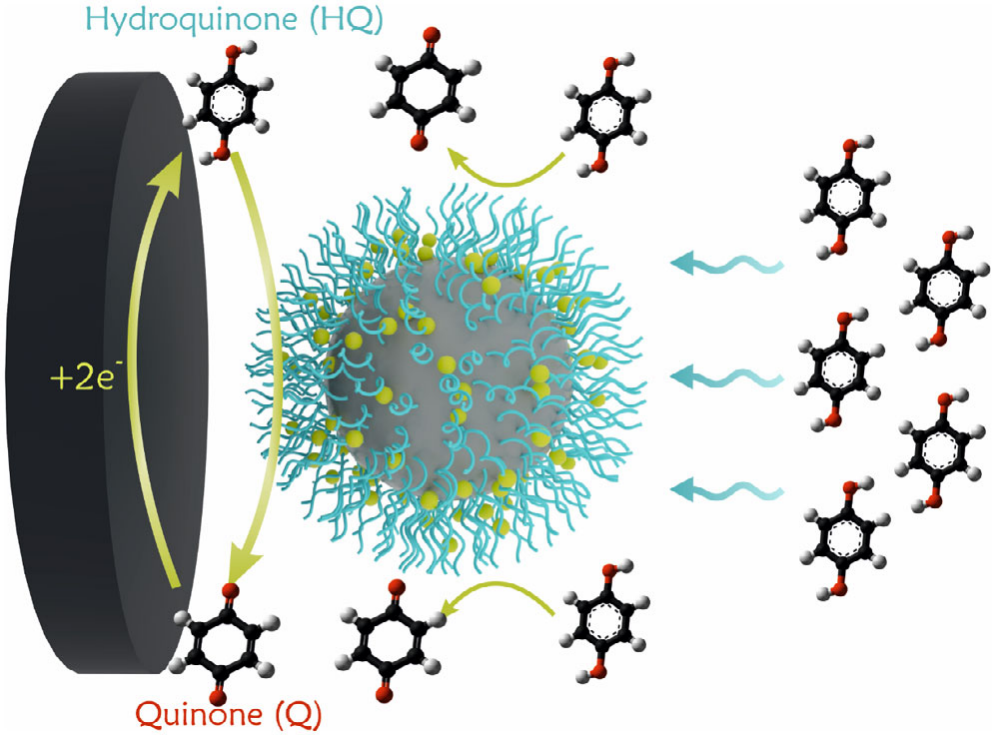
Schematic visualization of redox process occurring on laccase‐carrier modified working electrode.

HQ was selected as a model substrate to investigate the bioelectrocatalytic properties of the system. The detection mechanism is illustrated in Figure 4. HQ is catalytically oxidized by laccase immobilized within PDMAEMA brushes, resulting in the formation of 1,4‐benzoquinone (Q) and the reduced form of the enzyme (HQ → Q + 2H^+^ + 2e^−^). The generated quinone diffuses to electrode active sites and is subsequently electrochemically reduced, and the resulting current is proportional to the HQ concentration in solution (Q + 2H^+^ + 2e^−^ → HQ). The reduced laccase is reoxidized by molecular oxygen and is not consumed during the electrochemical cycle. Given that the oxygen concentration in air‐saturated water (∼260 µM) [26] significantly exceeds the Michaelis constant of laccase for oxygen (∼10 µM) and that copper oxidation is a spontaneous process, the rate‐limiting step is the diffusion of quinone to the electrode surface. The reduction current is used as the analytical signal.

**FIGURE 5:**
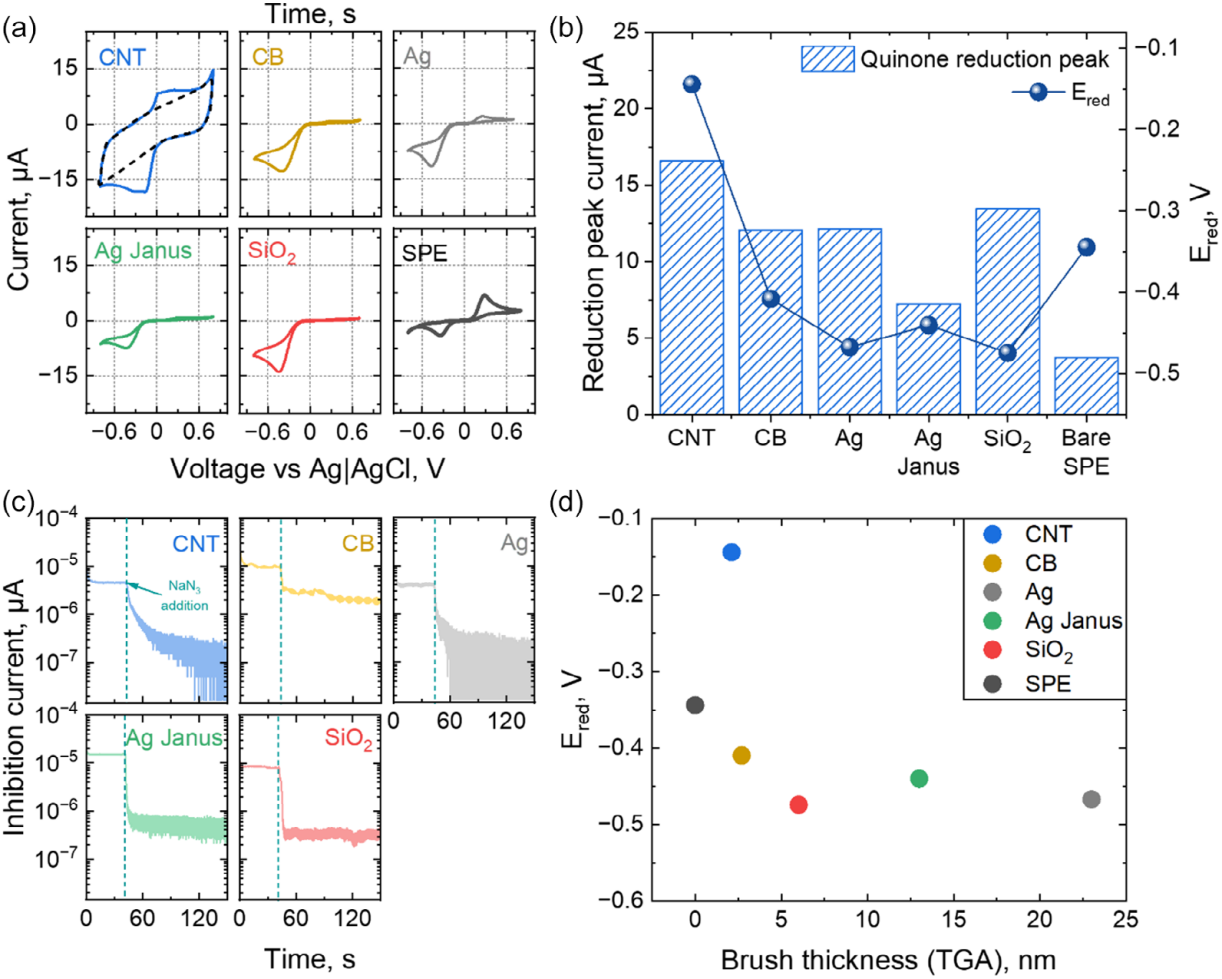
(a) Cyclic voltammetry curves of SPE and differently modified SPE electrodes in 0.1M HQ in 0.1M pH = 4 acetate buffer solution at 50 mV/s scan rate; (b) absolute values of reduction peak and reduction peak potential for differently modified SPE; (c) chronoamperometric curves at *V* = −0.6 V and under constant stirring obtained in the same solution, demonstrating drop of current during inhibition of enzymes by addition of a NaN_3_ solution; (d) reduction peak potential vs brush thickness of carrier (TGA).

To confirm that the observed redox reaction is enzyme‐catalyzed, we conducted an electrochemical investigation of the HQ/Q redox process using variously modified carbon SPEs. The CV curves obtained in a 0.1 mM HQ solution in 0.1 M acetate buffer at pH = 4 are presented in Figure 5a. All electrode modifications exhibit a catalytic CV shape, characterized by a significantly lower (as observed for CNT‐based systems) or a nearly absent anodic (oxidation) peak compared to the cathodic (reduction) peak. The amplified cathodic peak is attributed to the efficient catalytic oxidation of HQ by immobilized laccase, which generates a high local concentration of benzoquinone near the electrode surface while HQ is nearly absent. Control experiments using electrodes modified with (i) bare particles, (ii) enzyme‐immobilized particles, (iii) brush‐coated particles, and (iv) brush‐coated particles with immobilized laccase support this interpretation (Figures S9–S18): symmetrical CV curves in the absence of enzyme confirm the noncatalytic nature of the redox process. Additionally, CNT‐modified electrodes show a considerable capacitive current (dashed black line on Figure 5a), diminishing the relative contribution of faradaic current. This effect is likely due to the presence of exposed CNT surfaces not fully coated with PDMAEMA brushes, which results from difficulties in nanoparticle dispersion during synthesis. This leads to the observation of two reduction/oxidation peaks at different potentials. However, in some synthesis batches, the dispersibility of CNT could be good resulting in better brush coverage and lower capacitive current (Figure S12). Thus, the immobilization of enzymes in PDMAEMA brushes on any type of carrier results in high catalytic activity, with the HQ oxidation process at the electrode being significantly reduced and occurring almost entirely enzymatically, except in the case of CNT carriers.

Additionally, the electrochemical activity of the enzymes was confirmed by an inhibition test when the chronoamperometry (CA) was performed at E = −0.6V vs under constant stirring to provide mass transfer of the substrate to the electrode, followed by the addition of 100 µL of 0.1M NaN_3_ aqueous solution as an inhibitor to the system at *t* = 40 s (Figure 5c). Azide anions are strong ligands for enzyme's copper active centres. Therefore, the coupling of copper with $N_{3}^{-}$ results in a significant drop in the current. Thus, the PDMAEMA brush under this pH and polymer chain conformation provides conditions where the enzymes activity is elevated and the signal for HQ detection is significantly amplified.

The higher conductivity of carrier according to EIS data results in higher heterogeneous charge transfer constant and, as consequence, lower overpotential for the reduction of quinone (Figure 5b). Nonconductive SiO_2_, as well as conductive Ag, Ag‐Janus, and CB particles, that are completely covered with a brush, result in a higher reduction overpotential and peak separation value compared to bare SPE and CNT. It correlates with heterogeneous charge‐transfer constant values obtained in ferricyanide solution. We attribute it to brush insulating effect of such particles where the diffusion of the substrate to electrode is slower. For the conductive CNT, where the brush thickness is considerably smaller, and there is a presence of open surface, the peak potential, and peak–peak separation are smaller than those for the bare SPE. Thus, the conductive particles with small brushes lead to a more reversible nature of the redox process and enhance the charge transfer. However, the surface openness leads to higher capacitive current and less pronounced catalytical shape of the curve.

### 3.4 Sensor Parameters and Performance Comparison

**FIGURE 6:**
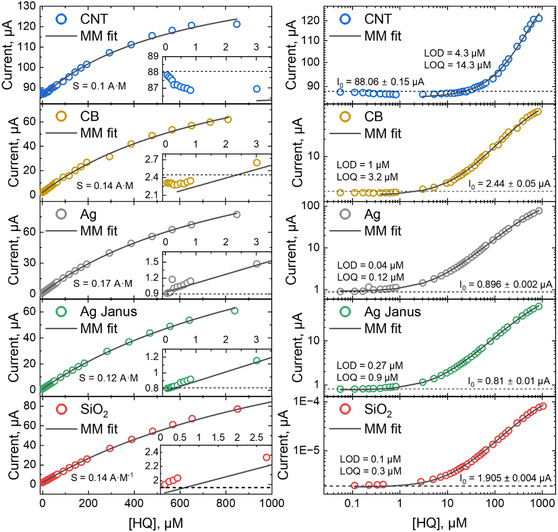
NPV calibration for HQ solutions of different concentrations in 0.1 M pH = 4 acetate buffer plotted in (i) lin–lin and (ii) log–log scale. The NPV was performed with 0.1 s pulse time, 1s sample period, and 10 mV step size. The dashed line shows the mean value of the blank buffer solution with its standard deviation.

For defining the sensor performance parameters, the calibration of the sensor was performed by three techniques: differential pulse voltammetry (DPV) (Figures S24–S29; Table S5), normal pulse voltammetry (NPV) (Figure 6, Table 5), and CA (Figures S22 and S23; Table S4). The electrodes were calibrated in 0.1 M pH = 4 acetate buffer with gradual addition of HQ solution to assess the main parameters such as LOD, sensitivity (*S*), and the detection range. The calibration by using CA is possible and demonstrated in Figures S22 and 23. However, for this technique, the LOD becomes very high (>1 µM) because capacitive current cannot be efficiently suppressed at short timescales, while at longer timescales, the Faradaic current is already too low once the capacitive component has decayed. Pulse voltammetry techniques are more sensitive to low concentration of analyte due to the effective suppression of the capacitive current from the signal, reducing the noise, and due to short time of current measurements, during which the amount of analyte does not drop significantly. Therefore, DPV and NPV will be discussed in more detail.

**TABLE 5:** Sensor parameters obtained from calibration curves measured by NPV.

	CNT	CB	Ag	Ag Janus	**SiO**_**2**_
S, A·M^−1^	0.1	0.14	0.17	0.12	0.14
LOD, µM	4.3	1	0.04	0.27	0.1
Detection range, µM	14.3–321	3.2–446	0.12–484	0.9–655	0.3–750

Note: Upper LOD range was determined as 0.5 × K_m_, and LOD was determined as 3·σ/S, where σ – standard deviation of background current (empty buffer). Lower LOD range was estimated as LOQ = 10·σ/S.

DPV is very effective at suppressing capacitive current, which is critical for highly conductive carbon‐based carriers. As a result, DPV should provide lower noise and lower LOD for these materials. NPV, in contrast, generally shows higher noise due to longer pulses. However, because NPV reaches a quasi‐steady state (where key parameters such as diffusion layer thickness, concentration gradient, and effective electron‐transfer rate stabilize), nonconductive SiO_2_ carriers, and silver carriers with thick brushes should exhibit a more extended linear range at higher concentrations, extending the upper detection limit.

The calibration curves obtained by both pulse voltammetry techniques follow the Michaelis–Menten kinetics with an $i_{0}$ offset according to Equation (8)

(8) $$i=i_{0}+\frac{i_{\max}\cdot \left[HQ\right]}{K_{m}+\left[HQ\right]}$$

where [*HQ*] is the HQ concentration, *i*_max_ is the maximum current density at a saturating substrate concentration for a given enzyme concentration, and *K*_*m*_ is the Michaelis constant. The sensitivity was determined as a slope in the linear region of the current vs concentration curve and is equal to $S=\frac{i_{\text{max}}}{K_{m}}$. The LOD is calculated as three times the standard deviation of the blank buffer signal divided by the sensitivity. Obtained parameters are illustrated in Table 5. The mean value of background current is denoted as *I*_0_ with calculated standard deviation. For all samples, a clear plateau is not observed, which we attribute to an abundance of enzymes and the unsaturation of the enzyme capacity even at large concentrations of HQ.

The CNT‐modified electrode demonstrated the poorest performance characteristics. Although the sensitivity of all sensors was comparable, the CNT‐based sensor exhibited the highest LOD, with values of 4.3 µM (NPV) and 1.7 µM (DPV). The other carriers showed similar performance in DPV calibration, with LODs ranging from 0.08 to 0.21 µM and sensitivities between 0.07 and 0.11 A·M^−1^. NPV calibration not only resulted in slightly higher sensitivity but also introduced greater noise, which consequently increased the LOD. An exception to this trend was the Ag‐carrier, which achieved the lowest observed LOD of 40 nM. Overall, carriers fully covered with polymer brushes yielded the best sensor performance – relatively high sensitivity combined with very low LODs (below 0.21 µM). In contrast, the higher conductivity and larger open surface area of CNTs did not lead to increased sensitivity; instead, the greater noise hindered the detection of low analyte concentrations.

**FIGURE 7:**
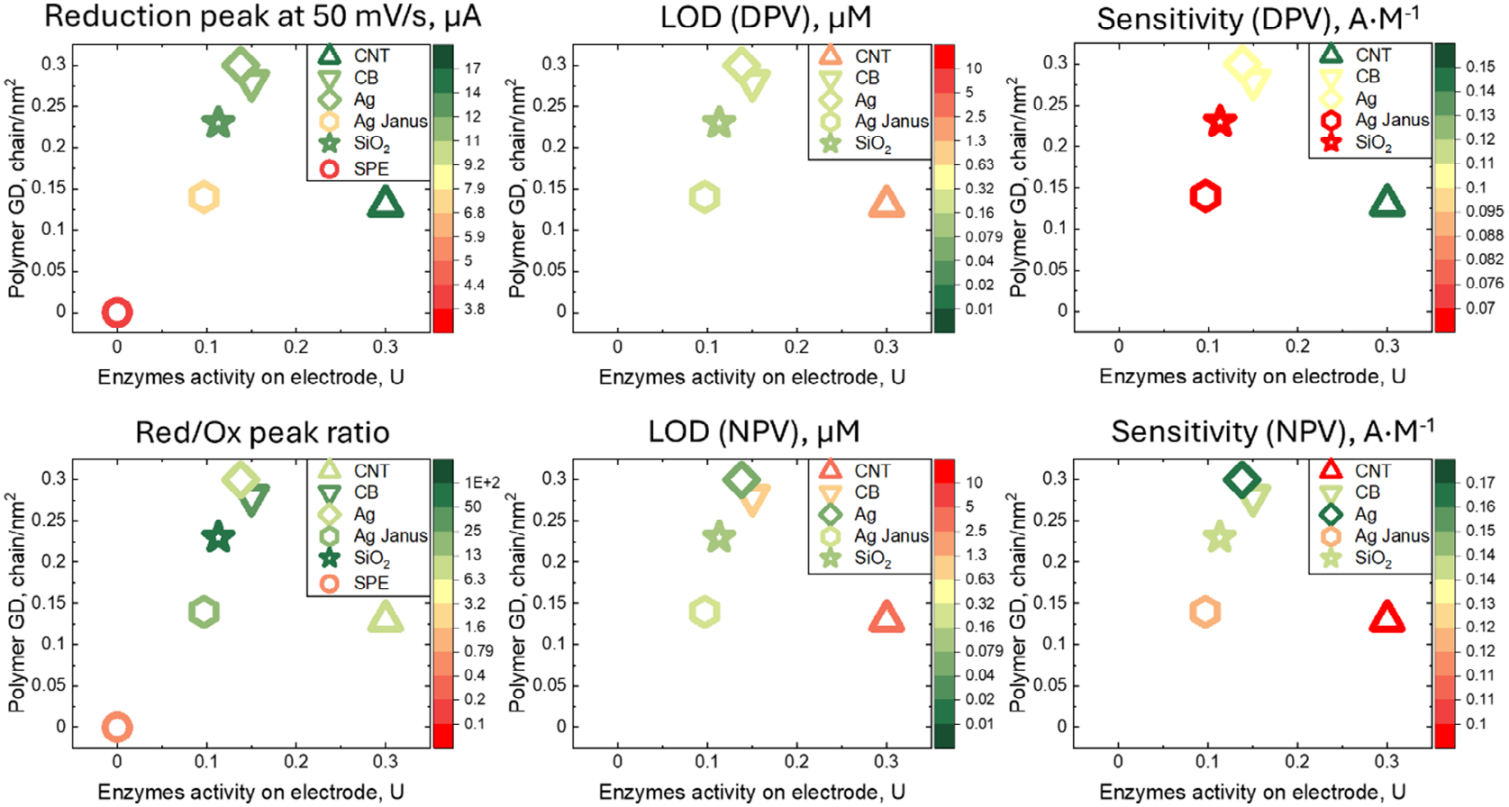
Map of sensor electrochemical properties as function of carriers polymer GD and their ability to immobilize enzyme.

To determine the difference in performance between carriers, maps of the main electrode characteristics were plotted in Figure 7. The main axes represent the GD of the polymer – which reflects the ratio between polymer thickness and its molecular weight – and the absolute activity of the enzymes responsible for the catalytic reaction. All carriers, except CNT, have similar GD and enzyme activity on electrode, which are in range of 0.23–0.3 chain·nm^−2^ and 0.1–0.15 U, respectively, whereas for CNTs GD is half as much (0.13 chain·nm^−2)^, but the absolute activity of the enzymes is twice as high (0.3 U). We propose the following scenario to explain different behaviour of sensors based on CNTs modified by PDMAEMA. Grafting of PDMAEMA brushes on CNT surface is very challenging since it is very difficult to disperse these particles. Therefore, the CNT carriers still possess open surface and entangled nature, creating a more conductive and percolated network on electrode surface. It results in a higher absolute reduction peak current. However, the Red/Ox peak ratio is not significant, which implies that the Faradaic current mediated by enzymes compared to the Faradaic current obtained on the open surface of the electrode. Moreover, the presence of open surface results in two reduction peaks on both linear CV and pulse voltammetry techniques (Figure S25), where at lower overpotential reduction occurs on open surface and at large overpotential on grafted or SPE electrode surface itself. For other carriers, although the amount of enzymes is twice as low as than that for CNT, it results in significantly higher reduction peak and Red/Ox peak ratio. The enzymes are active inside the PDMAEMA brush and effectively oxidize HQ. Thus, the fully covered carriers with PDMAEMA brush (CB, SiO_2_, Ag, Ag‐Janus) resulted in a higher catalytical activity of the enzymes and, as a consequence, a greater supression of HQ oxidation on the electrode surface at lower enzymes content compared to CNT carriers.

Thus, we can observe that the more conductive carrier and electrode surface does not always result in better sensor performance. The sensitivity for all carriers is within the range from 0.07 to 0.11 A·M^−1^ and is of the same order of magnitude, regardless of the brush thickness and enzyme amount. If the sensor is not going to be significantly miniaturized, the most important parameter in the end is the LOD. One aims to determine the lowest possible concentration quickly and precisely. When sensitivity values are similar across sensors, the LOD will primarily depend on the noise level. All sensors were measured under the same conditions on the same device; therefore, the dominant contribution to noise in this case is from the capacitive current.

The overall current can be described as the additive sum of capacitive (*i*_c_) and Faradaic (*i*_f_) currents as functions of time (Equation (9)):

(9) $$i(t)=i_{c}(t)+i_{f}(t)$$

For diffusion‐controlled systems, the Faradaic current is proportional to the active surface area, effective analyte concentration, and inversely proportional to the square root of time (Equation (10)):

(10) $$i_{f}=\frac{nFAC\sqrt{D}}{\sqrt{\pi t}}$$

The capacitive current depends on the applied potential, the overall system resistance, and the electrode capacitance and follows an exponential decay with time (Equation (11)):

(11) $$i_{с}=\frac{E}{R}e^{-\frac{t}{RC}}$$

Although the exponential decay is faster than the $t^{-1/2}$ decay of the Faradaic current, at short timescales, the pre‐exponential factor and the magnitude of the capacitance strongly influence the current. The simulation of *i*_f_ and *i*_c_ was performed based on the EIS and CV data obtained and discussed above (Figure S30). In the case of CNTs, which have a large surface area and are not completely covered with polymer brushes, this results in a low resistance and a high capacitance (as shown by EIS data). As a result, the capacitive current starts from a large initial value and decays relatively slowly, maintaining a higher noise level over time compared to other particles. Consequently, the Faradaic current does not dominate significantly over the capacitive current, explaining the high LOD values for CNT‐based sensors.

The next highest LOD was observed for the CB‐based carriers. Although CV data does not show significant capacitive currents, EIS measurements reveal a low charge transfer resistance, implying a relatively higher capacitive contribution to the overall current immediately after potential application. However, the ratio of *i*_c_ to *i*_f_ is much lower than that for CNT, resulting in better performance.

In contrast, other systems, such as Ag‐ and Ag‐Janus carriers with thick polymer brushes, and nonconductive SiO_2_ carriers, exhibit low noise levels. The capacitive current decays rapidly at short time scale. In these cases, the conductivity of the bare SPE is sufficient to support a decent Faradaic current, leading to much lower LOD values. That is why the more insulated carriers, such as the Ag‐carrier, led to the best performance given a sufficient conductivity of the carrier core and electrode surface.

**FIGURE 8:**
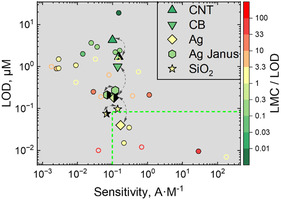
Ashby‐like plot for LOD vs sensitivity colour‐mapped by LMC/LOD ratio, where LMC is lowest measured concentration. Full and half‐full scatters correspond to NPV and DPV measurements techniques, respectively. Full circles correspond to laccase‐based sensors; empty circles correspond to laccase‐free sensors. The points correspond to references in Supplementary Information.

When comparing the performance of our sensors with published enzymatic and nonenzymatic sensors for HQ/quinone detection, we found that comparing them directly is quite difficult because experiments were performed under different conditions, different activity of enzymes, scanning speeds, sample sizes, acquisition times for data averaging to reduce noise and not always all data are available for comparison. We considered the following parameters as criteria for benchmarking: sensitivity, LOD, and detection range (Figure 8). In many cases, the LOD was obtained by extrapolation, and the further the LOD value obtained from the lowest measured concentration (LMC), the less reliable it is. Therefore, we introduced the ratio between the LMC and the LOD, LMC/LOD, as a quality criterion for the results. Ideally, the LMC/LOD should be less than one so that a transition from the measured to the unmeasured range is observed. LMC/LOD is greater than 10 indicates that the LOD value is not highly reliable.

The sensors we used are within the reliable region. The comprehensive analysis of the other research papers, which demonstrated allegedly better performance, was performed in our previous research. Plotting the Ashby‐like plot, the sensors based on SiO_2_ and Ag carriers fall within the range of the most sensitive sensors, exhibiting one of the lowest limits of detection for HQ detection. Enzyme‐free sensors typically have higher sensitivity and lower LOD that is due to fast kinetics of reaction not limited by diffusion that allows high current. These sensors however have issues with selectivity, whereas enzyme‐based biosensors allow selective detection.

## 4 Conclusion

In this work, we questioned the necessity of conductive materials with a large surface area for the design of working electrodes for enzymatic electrochemical biosensors. In addition to the previously studied insulating silica, conductive CNTs, CB, silver, and silver‐Janus particles were used as core materials. All these carriers offer several advantages: (i) high enzyme loading; (ii) high catalytic activity of the enzyme, significantly suppressing HQ oxidation at the electrode surface; (iii) controllable and quantifiable enzyme immobilization; (iv) low limits of detection (LOD); (v) relatively high sensitivity; and (vi) a significantly broad detection range. The choice of core material did not result in a significant change in sensor sensitivity. However, the nature of the carrier surface strongly affected the electrode LOD, and a high electrode conductivity did not lead to better performance. For instance, although loosely grafted CNTs exhibit high conductivity and consequently low overpotential for HQ reduction, the high capacitive current diminishes the contribution of the Faradaic current, resulting in a low signal‐to‐noise ratio. Thus, the polymer brush is a key element not only for ensuring high catalytic activity of the enzymes but also for improving the signal‐to‐noise ratio. Overall, the performance of the developed sensors was compared to other laccase‐based sensors for HQ detection reported in the literature. The best performance was demonstrated by silver‐based carriers, with a sensitivity of 0.17 A·M^−1^, an LOD of 0.04 µM, and a detection range of 0.12–484 µM, outperforming most existing materials under the conditions under which they were tested.

## Supporting Information

Additional supporting information can be found online in the Supporting Information section. **Supporting Figure S** **1**: Thermogravimetry (TGA) measurements of different hairy carriers: a) CNT, b) CB, c) Ag, d) Ag Janus carriers. The TGA measurements were conducted at heating rate 10 K/min in nitrogen atmosphere. **Supporting Figure S2a**: TGA analysis of different batches of SiO_2_ modified with PDMAEMA brushes. The degradation of PDMAEMA starts around 200°C. **Supporting Figure S2b**: TGA analysis of different batches of CB modified with PDMAEMA brushes. The degradation of PDMAEMA starts around 200°C. **Supporting Figure S2c**: TGA analysis of different batches of CNT modified with PDMAEMA brushes. The degradation of PDMAEMA starts around 200°C. **Supporting Figure S2d**: TGA analysis of different batches of Ag modified with PDMAEMA brushes. The degradation of PDMAEMA starts around 200°C. **Supporting Figure S2e**: TGA analysis of different batches of Ag‐Janus modified with PDMAEMA brushes. The degradation of PDMAEMA starts around 200°C **Supporting Figure S3**: Zeta‐potential measurement as a function of pH for (a) bare (CNT, CB, Ag, and SiO_2_) and (b) PDMAEMA modified carriers (CNT, CB, Ag, Ag‐Janus, and SiO_2_). c) SEM images of PDMAEMA modified various carriers (CNT, CB, Ag, Ag‐Janus, and SiO_2_). Scale bar is 500 nm. **Supporting Figure S4**: Zeta‐potential measurement as a function of pH for bare, Br‐initiator modified, PMAEMA‐brush modified carriers: a) CNT; b) CB; c) SiO_2_; d) Ag and Ag Janus. **Supporting Figure S5**: Scheme of enzyme immobilization on/onto hairy carrier as well as dependency of immobilized enzymes activity on initial activity of enzymes in buffer. **Supporting Figure S6**: Colour maps of plot GD vs TGA brush thickness for enzyme activity per a) mg of polymer and b) mg of enzymes; Colour maps of plot M_w_ vs TGA brush thickness for enzyme activity per c) mg of polymer and d) mg of enzymes. The surface area of Janus‐particles was taken as a value of whole particle surface area. **Supporting Figure S7**: Peak‐peak distance vs brush thickness obtained from CV measurements in solution containing 5 mM K_3_[Fe(CN)_6_] and 0.1 M KCl; scan rate 50 mV/s. **Supporting Figure S8**: Equivalent electrical circuit used for EIS data fitting. **Supporting Figure S9**: Bode plot of SPE and SPE modified electrodes obtained in 5 mM K_3_[Fe(CN)_6_] and 0.1 M KCl solution at formal potential offset with 10 mV amplitude. **Supporting Figure S10**: Z_mod_ at low frequency vs frequency at phase shift minimum obtained from Bode plot of EIS measurements in 5 mM K_3_[Fe(CN)_6_] and 0.1 M KCl solution at formal potential offset with 10 mV amplitude. **Supporting Figure S11**: CVA curves for SiO_2_ carriers of different modifications‐based electrode measured in 0.1 mM hydroquinone solution in 0.1M pH = 4 acetate buffer at different scan rates: a) pristine SiO_2_, b) SiO_2_ – Laccase; c) SiO_2_ – PDMAEMA; d) SiO_2_ – PDMAEMA ‐ Laccase[1]. **Supporting Figure S12**: Reduction peak value vs square route of scan rate and its linear fit for SiO_2_ carriers of different modifications‐based electrode measured in 0.1 mM hydroquinone solution in 0.1M pH = 4 acetate buffer [1]. **Supporting Figure S13**: a) CV of differently modified electrodes measured in 0.1 mM hydroquinone solution in 0.1M pH=4 acetate buffer at 100 mV/s; b) Quinone reduction peak values taken from CV results; c) chronoamperometry measurements at constant stirring with addition of NaN_3_ solution as inhibitor; d) CV of SiO_2_ ‐ PDMAEMA – Laccase electrode before and after inhibition (measured at 100 mV/s) [1]. **Supporting Figure S14**: CV curves for CNT carriers of different modifications‐based electrode measured in 0.1 mM hydroquinone solution in 0.1M pH = 4 acetate buffer at different scan rates: a) pristine CNT, b) CNT – Laccase; c) CNT – PDMAEMA; d) CNT – PDMAEMA – Laccase. **Supporting Figure S15**: (i) CV in 0.1mM hydroquinone solution in 0.1M pH = 4 acetate buffer and (ii) inhibition by 0.1M NaN_3_ solution of CNT‐PDMAEMA‐Laccase based sensor with grafting density 100%. **Supporting Figure S16**: a) CV of differently modified electrodes measured in 0.1 mM hydroquinone solution in 0.1M pH = 4 acetate buffer at 100 mV/s; b) Quinone reduction an oxidation peak values taken from CV results; c) chronoamperometry measurements at constant stirring with addition of NaN_3_ solution as inhibitor; d) CVA of CB ‐ PDMAEMA – Laccase electrode before and after inhibition (measured at 100 mV/s). **Supporting Figure S17**: CV curves for Ag‐Janus carriers of different modifications‐based electrode measured in 0.1 mM hydroquinone solution in 0.1M pH = 4 acetate buffer at different scan rates: a) pristine Ag‐Janus, b) Ag‐Janus – Laccase; c) Ag‐Janus – PDMAEMA; d) Ag‐Janus – PDMAEMA – Laccase. **Supporting Figure S18**: a) CV of differently modified electrodes measured in 0.1 mM hydroquinone solution in 0.1M pH = 4 acetate buffer at 100 mV/s; b) Quinone reduction peak values taken from CV results; c) chronoamperometry measurements at constant stirring with addition of NaN_3_ solution as inhibitor; d) CV of Ag‐Janus ‐ PDMAEMA – Laccase electrode before and after inhibition (measured at 100 mV/s). **Supporting Figure S19**: CV curves for SiO_2_ carriers of different modifications‐based electrode measured in 0.1 mM hydroquinone solution in 0.1M pH = 4 acetate buffer at different scan rates: a) pristine Ag, b) Ag – Laccase; c) Ag – PDMAEMA; d) Ag – PDMAEMA – Laccase. **Supporting Figure S20**: a) CV of differently modified electrodes measured in 0.1 mM hydroquinone solution in 0.1M pH = 4 acetate buffer at 100 mV/s; b) Quinone reduction peak values taken from CV results; c) chronoamperometry measurements at constant stirring with addition of NaN_3_ solution as inhibitor; d) CV of Ag ‐ PDMAEMA – Laccase electrode before and after inhibition (measured at 100 mV/s). **Supporting Figure S21**: CV of differently modified SPE electrode at various scan rates in 0.1 mM HQ solution in 0.1 M acetic buffer pH = 4. **Supporting Figure S22**: a) Reduction peak of quinone vs log(v) obtained by CV measurements in 0.1mM HQ solution in 0.1 M acetic buffer pH = 4 at different scan rate (*v*), mV/s for differently modified SPEs; b) Quinone reduction peak value vs *v* ^1/2^ obtained during the same measurements. **Supporting Figure S23**: a) reduction peak potential and b) Red/Ox peak ratio vs. brush thickness (TGA). The values obtained from CV measurements of electrodes in 0.1M HQ in 0.1M pH = 4 acetate buffer solution at 50 mV/s. **Supporting Figure S24**: Chronoamperometric curves obtained in different concentrations of hydroquinone solution in 0.1M pH = 4 acetate buffer for differently modified SPEs. Data for SiO_2_ are taken from our previous study [1]. **Supporting Figure S25**: Calibration curves of a) CNT, b) CB, c) SiO_2__[1]_, d) Ag, e) Ag Janus carriers modified SPEs with immobilised laccase obtained by chronoamperometry in HQ solutions in 0,1M pH = 4 acetate buffer at potential E = ‐0.6 V vs Ag|AgCl electrode. The points correspond to current at t = 2s. The calibration curve for all carriers follows Michaelis‐Menten equation. Supporting Table S4. Parameters of sensors obtained via calibration using chronoamperometry technique at E = ‐0.6 V at various HQ concentration in 0.1M pH = 4 acetic buffer. **Supporting Figure S26**: DPV calibration for HQ solutions of diﬀerent concentrations in 0.1 m pH = 4 acetate buﬀer plotted in (a) lin‐lin and (b) log‐log scale. The DPV was performed with 0.1 s pulse time, 1s sample period, 10 mV step size, and 200 mV pulse. Dash – line shows themean value of the blank buﬀer solution with its standard deviation. Data for SiO_2_ are taken from our previous study [1]. **Supporting Figure S27**: CNT – PDAMAEMA carrier: a) Normal pulse voltammetry (NPV), b) differential pulse voltammetry (DPV) in log‐lin scale and c) DPV in lin‐lin scale for (i) HQ solutions of different concentrations in 0.1M pH = 4 acetate buffer and (ii) for pure acetate buffer. The NPV was performed with 0.1s pulse time, 1s sample period and 10 mV step size. The DPV was performed with 0.1s pulse time, 1s sample period, 10mV step size and 200 mV pulse. **Supporting Figure S28**: CB – PDAMAEMA carrier: a) Normal pulse voltammetry (NPV), b) differential pulse voltammetry (DPV) in log‐lin scale and c) DPV in lin‐lin scale for (i) HQ solutions of different concentrations in 0.1M pH = 4 acetate buffer and (ii) for pure acetate buffer. The NPV was performed with 0.1s pulse time, 1s sample period and 10 mV step size. The DPV was performed with 0.1s pulse time, 1s sample period, 10mV step size and 200 mV pulse. **Supporting Figure S29**: Ag – PDAMAEMA carrier: a) Normal pulse voltammetry (NPV), b) differential pulse voltammetry (DPV) in log‐lin scale and c) DPV in lin‐lin scale for (i) HQ solutions of different concentrations in 0.1M pH = 4 acetate buffer and (ii) for pure acetate buffer. The NPV was performed with 0.1s pulse time, 1s sample period and 10 mV step size. The DPV was performed with 0.1s pulse time, 1s sample period, 10 mV step size and 200 mV pulse. **Supporting Figure S30**: Ag Janus – PDAMAEMA carrier: a) Normal pulse voltammetry (NPV), b) differential pulse voltammetry (DPV) in log‐lin scale and c) DPV in lin‐lin scale for (i) HQ solutions of different concentrations in 0.1M pH = 4 acetate buffer and (ii) for pure acetate buffer. The NPV was performed with 0.1s pulse time, 1s sample period and 10 mV step size. The DPV was performed with 0.1s pulse time, 1s sample period, 10mV step size and 200 mV pulse. **Supporting Figure S31**: SiO_2_ – PDAMAEMA carrier [1]: a) Normal pulse voltammetry (NPV), b) differential pulse voltammetry (DPV) in log‐lin scale and c) DPV in lin‐lin scale for (i) HQ solutions of different concentrations in 0.1M pH = 4 acetate buffer and (ii) for pure acetate buffer. The NPV was performed with 0.1s pulse time, 1s sample period and 10 mV step size. The DPV was performed with 0.1s pulse time, 1s sample period, 10mV step size and 200 mV pulse. **Supporting Figure S32**: The capacitive and Faradaic current simulations for bare and differently modified SPE. The Faradaic current was calculated according to the Cotrell equation $i_{f}\frac{nFAC\sqrt{D}}{\sqrt{\pi t}}$, where n – number of electrons, F – Faradaic constant C·mol^−1^, A – active surface area m^2^, C – concentration of analyte mol·m^−3^, D – diffusion coefficient m^2^·s^−1^ (2·10^−10^ m^2^·s^−1^ for hydroquinone, t – time. The active surface area was taken from Supporting Table S1. Concentration of analyte was taken as bulk concentration – 0.1 mM (however, the local concentration of quinone can be higher than in bulk). The capacitive current was estimated as $i_{c}=\frac{E}{R}e^{‐\frac{t}{RC}}$, where E – applied potential, *C* – capacitance and R – corresponding resistance. If the EIS results were fitted with two R‐CPE elements, additive function of 2 exponents were used. The corresponding capacitance C was recalculated according to the equation C = (Y·R^1‐a^)^1/a^, where *Y* – CPE coefficient, *a* – CPE exponent. **Supporting Table S1**: Fitting parameters for obtained EIS spectra in 5 mM K_3_[Fe(CN)_6_] + 0.1M KCl solution. The offset voltage was equal to formal potential of the redox couple [Fe(CN)_6_]^3‐/4‐^ with a 10 mV voltage amplitude. **Supporting Table S2**: Values for calculation of active surface area, charge transfer resistance R_ct_ and heterogeneous rate constant k_0_ of electrodes for SPE and differently modified SPEs by various carriers. The active surface area values are obtained from CV data, measured in in 5 mM K_3_[Fe(CN)_6_] + 0.1M KCl solution by fitting the reduction peak value by Randles‐Sevcik equation. **Supporting Table S3**: Values for calculation of active surface area of electrodes for SiO_2_ particles of different modification [1]. **Supporting Table S4**: Parameters of sensors obtained via calibration using chronoamperometry technique at E = ‐0.6 V at various HQ concentration in 0.1 M pH = 4 acetic buffer. Supporting Table S5. Sensor parameters obtained from calibration curves measured by DPV.

## Author Contributions

All authors have given approval to the final version of the manuscript.

## Funding

This study was supported by Deutsche Forschungsgemeinschaft (Grant SY125/15‐1 and IO 68/20‐1), and Bundesministerium für Bildung und Forschung (Grant JaBaS FKZ: 031B1118Teil B).

## Conflicts of Interest

The authors declare no conflicts of interest.

## Supporting information

Supplementary Material

## Data Availability

The data that support the findings of this study are available from the corresponding author upon reasonable request.
