# Supplementary material for: Electrochemical Biosensor Based on Hairy Core–Shell Particles: Effect of Core Conductivity
Source: Small Sci. 2026 Apr 7;6(4):e202500550. doi: 10.1002/smsc.202500550 (PMC13063792; doi:10.1002/smsc.202500550)
Supplement: Supplementary file 1 — Supplementary Material [file SMSC-6-e202500550-s001.pdf]

Electrochemical Biosensor Based on Hairy Core-Shell Particles: Effect of Core Conductivity

Supporting information

Pavel Milkin[1], Anila Antony[2], Hongtao Cai[2], Ceyda Topal[1], Antonia Debevc[1], Anne Linhardt[2], Alla Synytska[2], Leonid Ionov[1*]*

Pavel Milkin and Anila Antony contributed equally to this work

[1]Faculty of Engineering Sciences, University of Bayreuth, Ludwig Thoma Str. 36A, 95447 Bayreuth, Germany

E-mail: leonid.ionov@uni-bayreuth.de, ORCID: https://orcid.org/0000-0002-0770-6140

[2]Functional Polymer Interfaces Group, Bayerisches Polymerinstitut (BPI), Universität Bayreuth, Universitätsstraße 30, 95447 Bayreuth, Germany

E-mail: alla.synytska@uni-bayreuth.de, ORCID: https://orcid.org/0000-0002-0643-7524

Carrier synthesis and enzyme immobilization

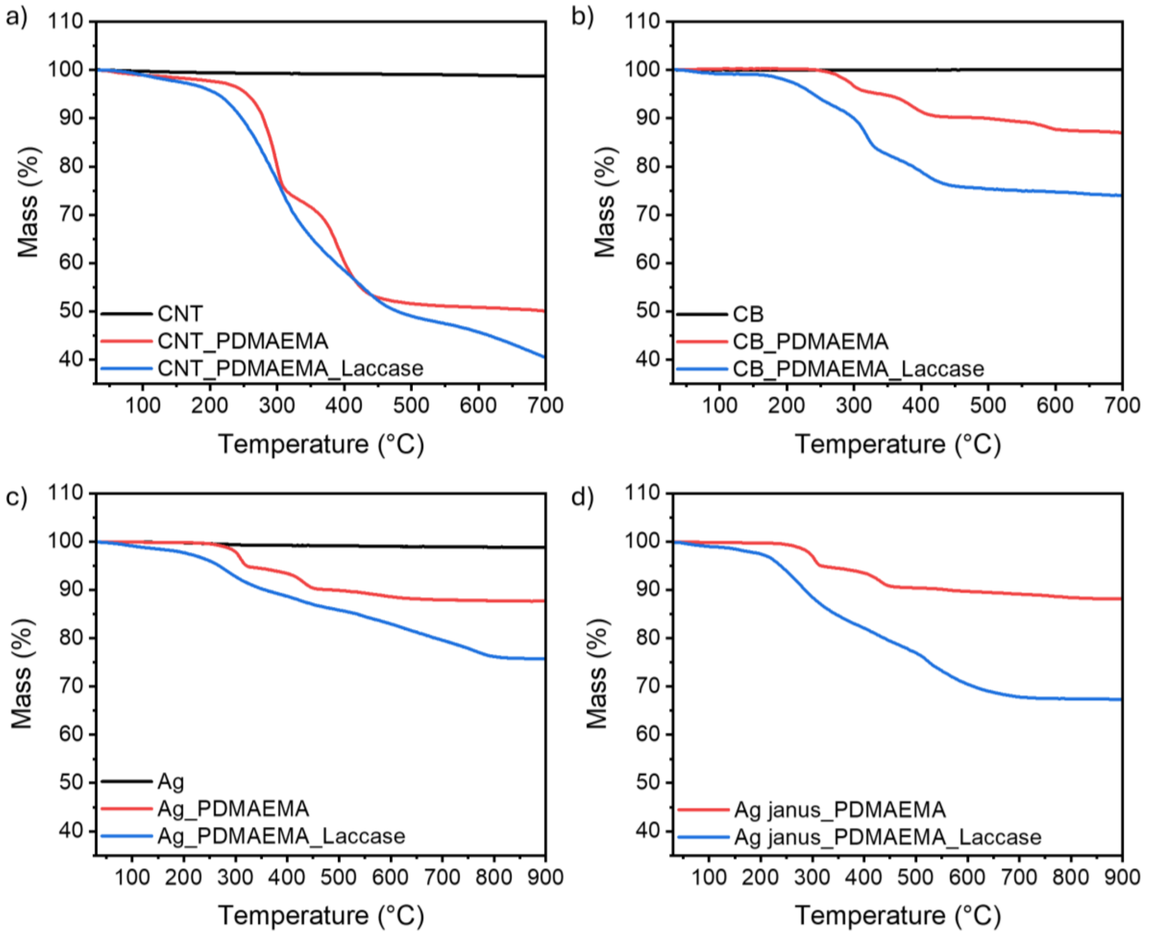

Figure S1. Thermogravimetry (TGA) measurements of different hairy carriers: a) CNT, b) CB, c) Ag, d) Ag Janus carriers. The TGA measurements were conducted at heating rate 10 K/min in nitrogen atmosphere.

Carrier	φ of core, wt. %	φ of brush, wt. %	φ of enzymes, wt. %
**CNT**	45.5	40.2	14.3
**CB**	76.2	11.6	12.2
**Ag**	78.9	9.9	11.2
**Ag Janus**	70.1	3.1	26.8
**SiO_2**	86.9	7.9	5.2

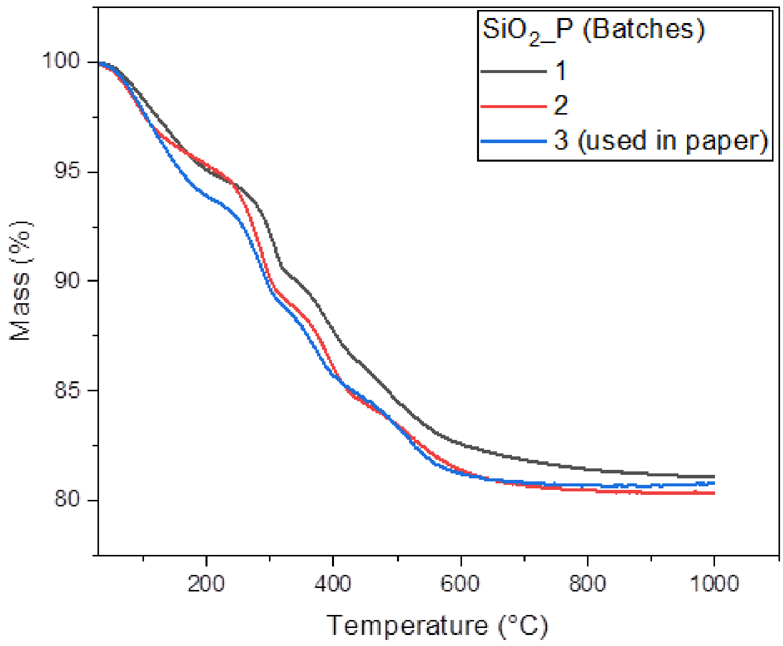

Figure S2a. TGA analysis of different batches of SiO_2 modified with PDMAEMA brushes. The degradation of PDMAEMA starts around 200 °C.

The polymer mass fraction according to TGA results for different batches of synthesis of SiO_2 – PDMAEMA carriers

SiO_2-PDMAEMA Batch Number	Mass fraction, %	Brush thickness (nm)^{TGA}
1	9.17	5.8
2	10.12	6.4
3 (used in the paper)	8.31	5.22

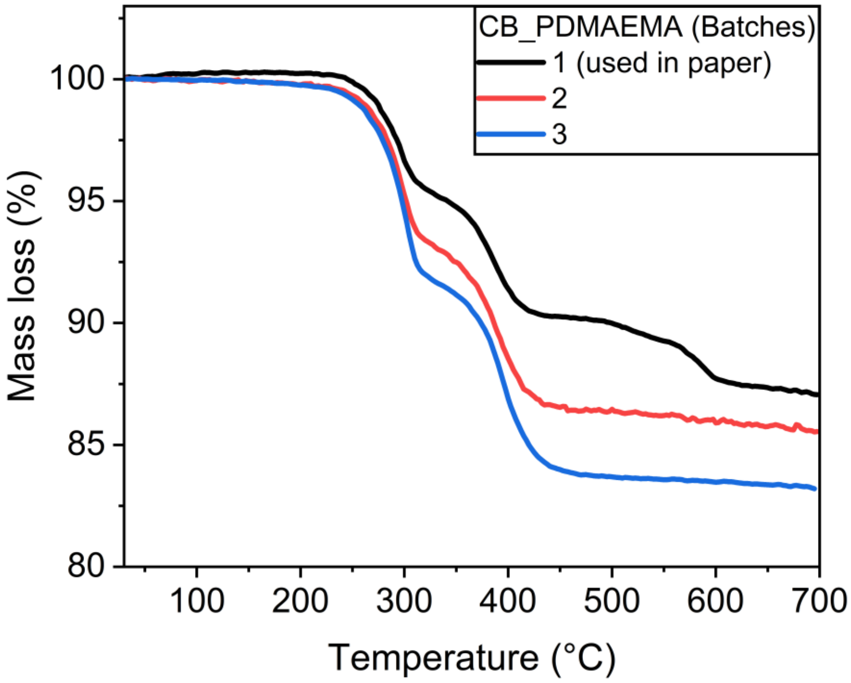

Figure S2b. TGA analysis of different batches of CB modified with PDMAEMA brushes. The degradation of PDMAEMA starts around 200 °C.

The polymer mass fraction according to TGA results for different batches of synthesis of CB – PDMAEMA carriers

CB-PDMAEMA Batch Number	Mass fraction, %	Brush thickness (nm)^{TGA}
1 (used in paper)	13.0	1.4
2	14.3	1.6
3	16.6	1.9

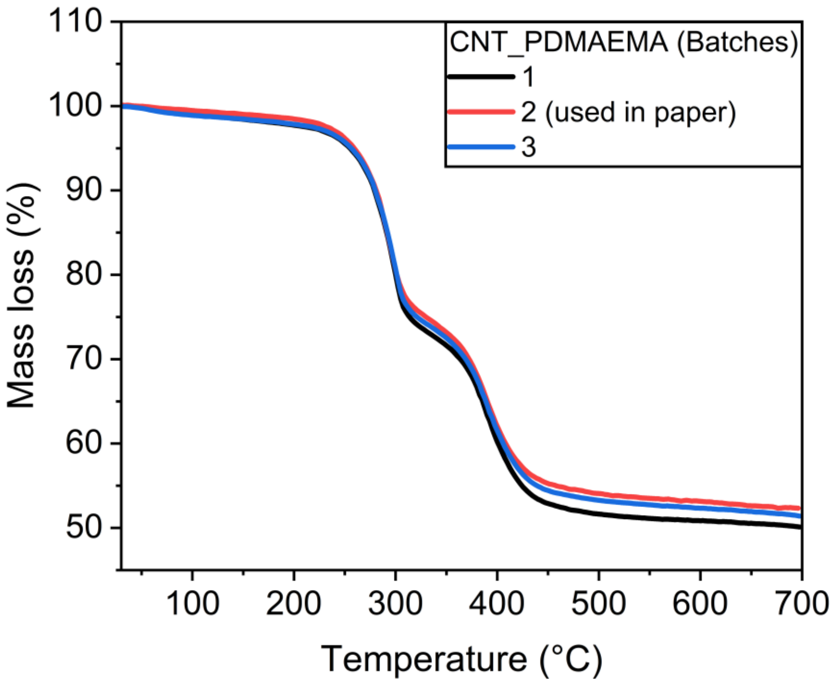

Figure S2c. TGA analysis of different batches of CNT modified with PDMAEMA brushes. The degradation of PDMAEMA starts around 200 °C.

The polymer mass fraction according to TGA results for different batches of synthesis of CNT – PDMAEMA carriers

CNT-PDMAEMA Batch Number	Mass fraction, %	Brush thickness (nm)^{TGA}
1	48.7	2.8
2 (used in paper)	46.8	2.7
3	47.5	2.7

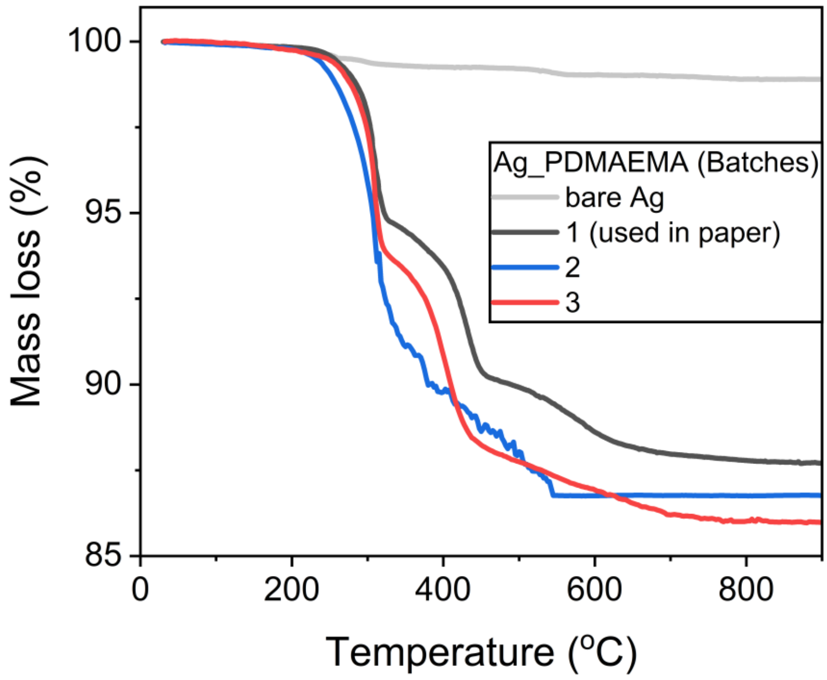

Figure S2d. TGA analysis of different batches of Ag modified with PDMAEMA brushes. The degradation of PDMAEMA starts around 200 °C

The polymer mass fraction according to TGA results for different batches of synthesis of Ag – PDMAEMA carriers

Ag-PDMAEMA Batch Number	Mass fraction, %	Brush thickness (nm)^{TGA}
1 (used in paper)	9.5	23
2	10.4	24
3	11.7	37

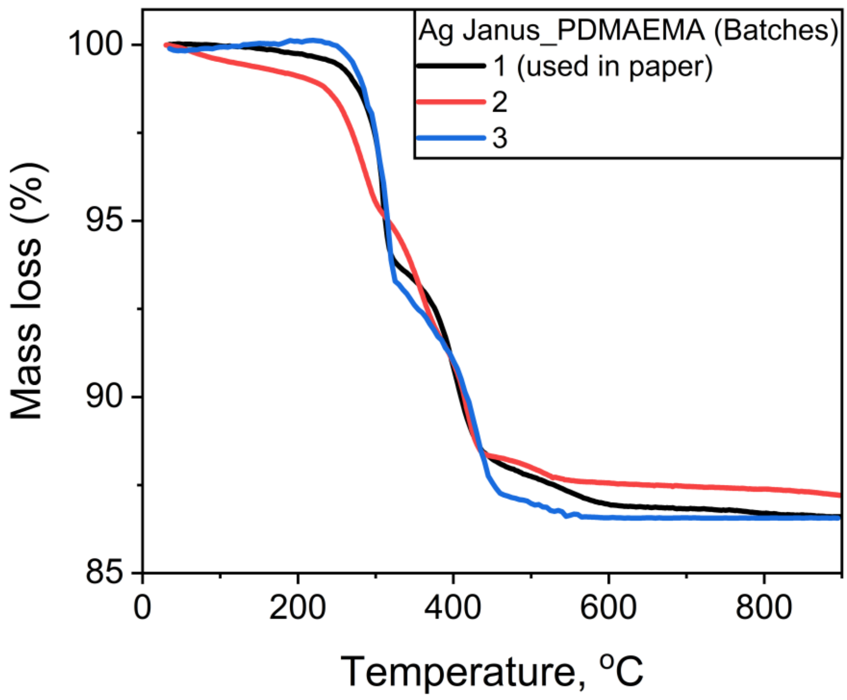

Figure S2e. TGA analysis of different batches of Ag-Janus modified with PDMAEMA brushes. The degradation of PDMAEMA starts around 200 °C

The polymer mass fraction according to TGA results for different batches of synthesis of Ag-Janus – PDMAEMA carriers

Ag-Janus-PDMAEMA Batch Number	Mass fraction, %	Brush thickness (nm)^{TGA}
1 (used in paper)	10.5	12.3
2	9.4	11.2
3	11.1	12.9

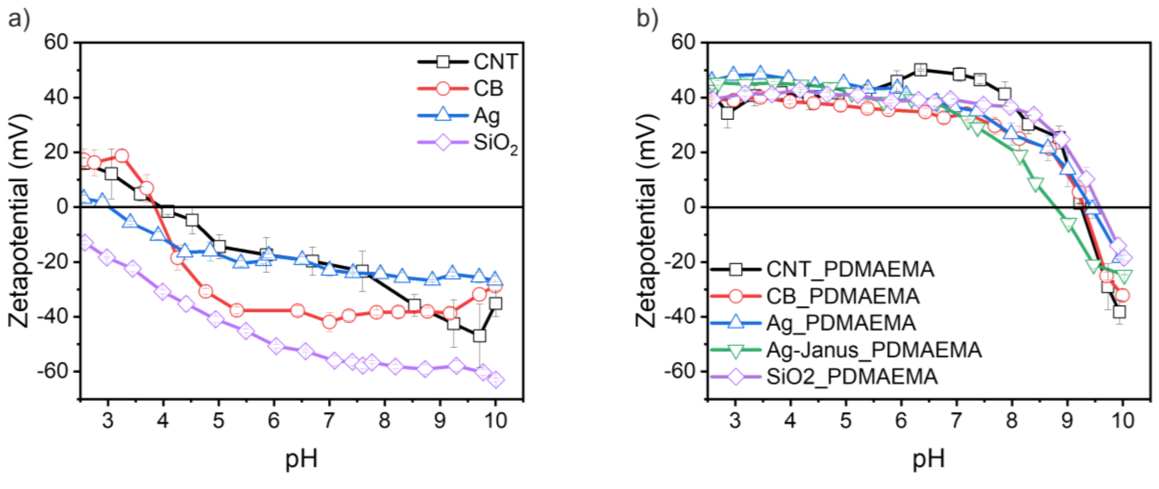

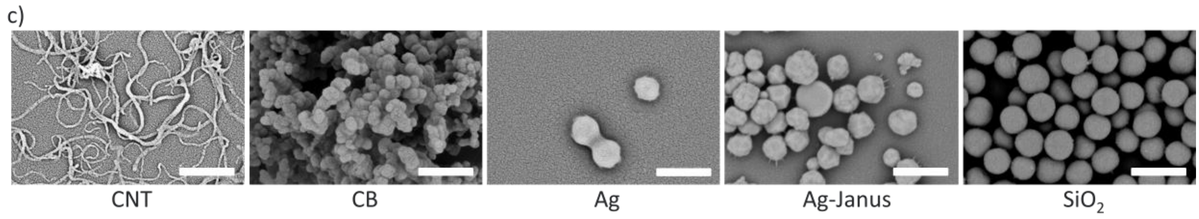

Figure S3. Zeta-potential measurement as a function of pH for (a) bare (CNT, CB, Ag, and SiO_2) and (b) PDMAEMA modified carriers (CNT, CB, Ag, Ag-Janus, and SiO_2). c) SEM images of PDMAEMA modified various carriers (CNT, CB, Ag, Ag-Janus, and SiO_2). Scale bar is 500 nm.

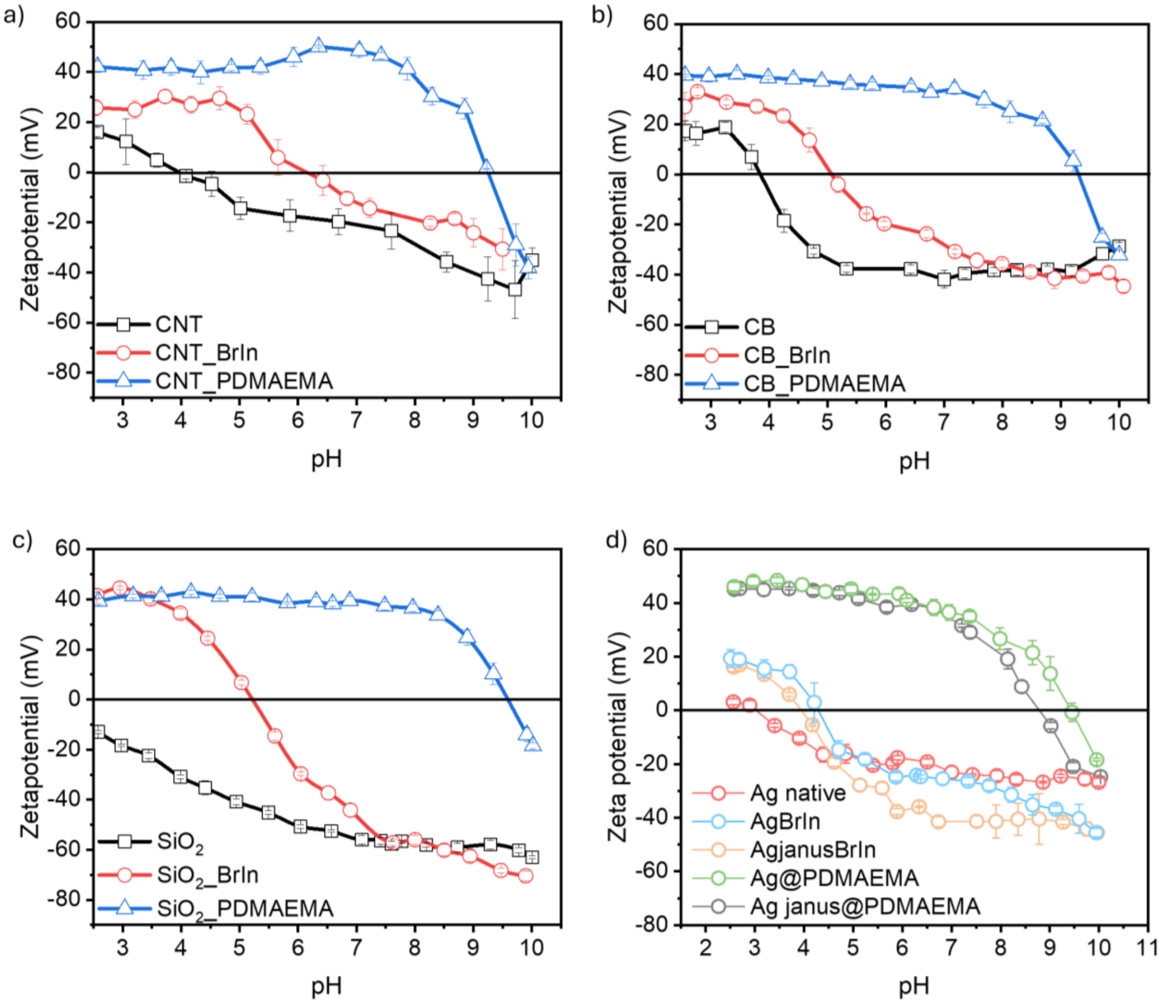

Figure S4. Zeta-potential measurement as a function of pH for bare, Br-initiator modified, PMAEMA-brush modified carriers: a) CNT; b) CB; c) SiO_2; d) Ag and Ag Janus

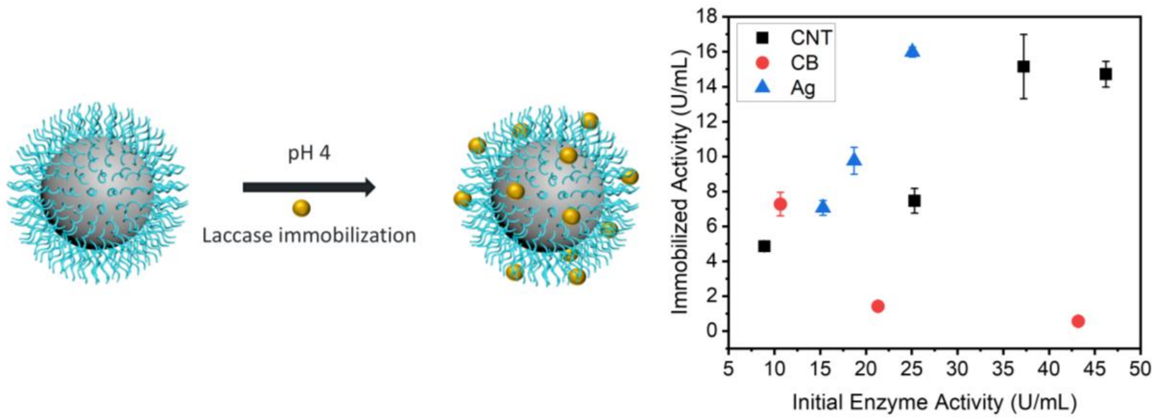

Figure S5. Scheme of enzyme immobilization on/onto hairy carrier as well as dependency of immobilized enzymes activity on initial activity of enzymes in buffer.

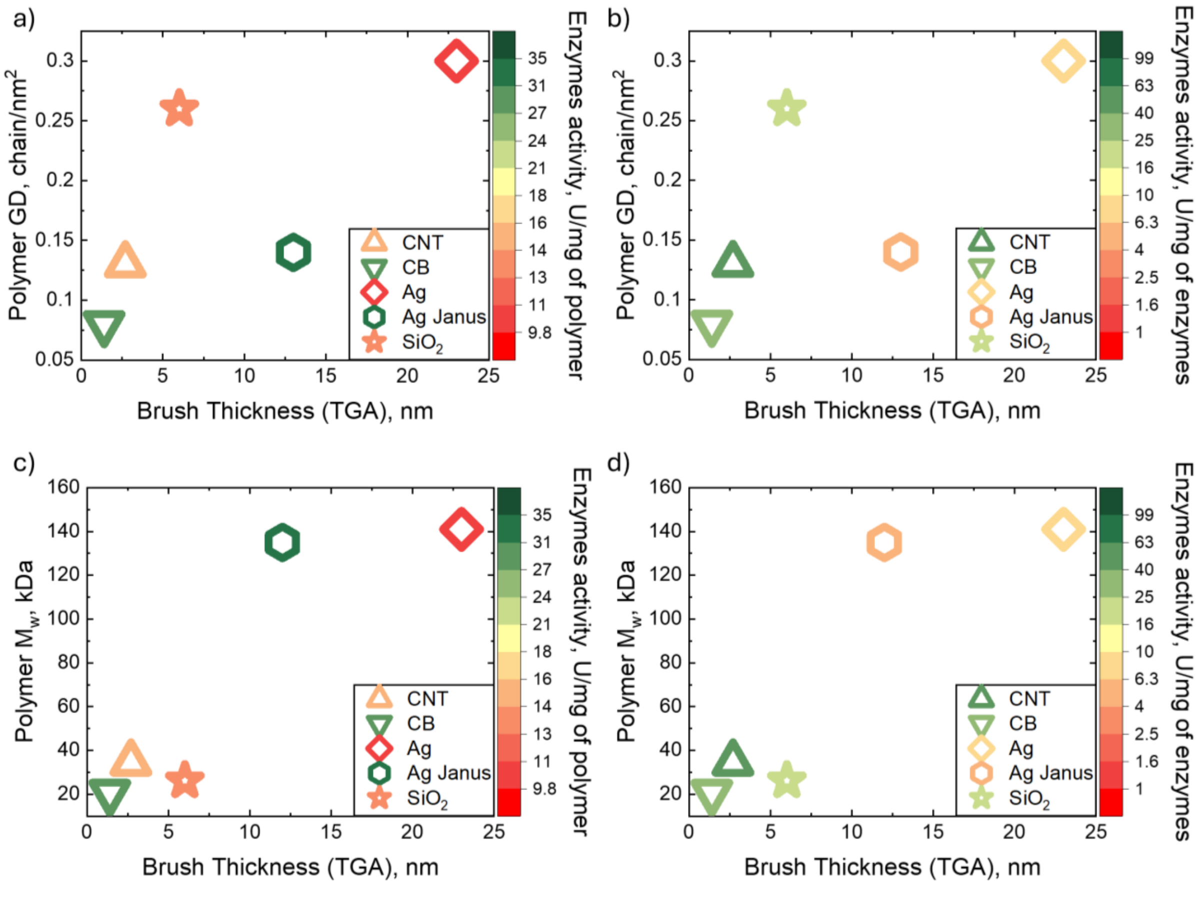

Figure S6. Colour maps of plot GD vs TGA brush thickness for enzyme activity per a) mg of polymer and b) mg of enzymes; Colour maps of plot M_w vs TGA brush thickness for enzyme activity per c) mg of polymer and d) mg of enzymes. The surface area of Janus-particles was taken as a value of whole particle surface area.

Electrochemical characterisation of electrodes

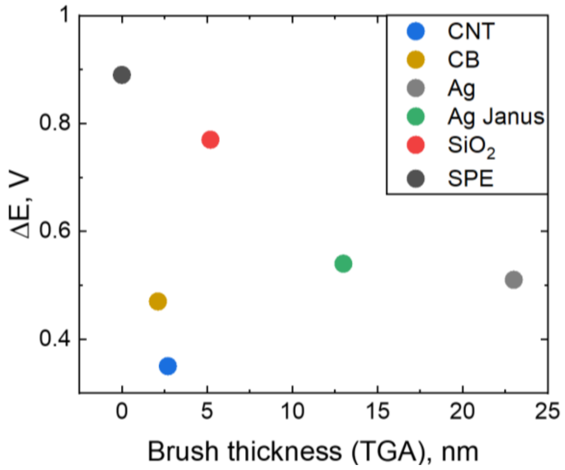

Figure S7. Peak-peak distance vs brush thickness obtained from CV measurements in solution containing 5 mM $K_3[Fe(CN)_6]$ and 0.1 M KCl; scan rate 50 mV/s

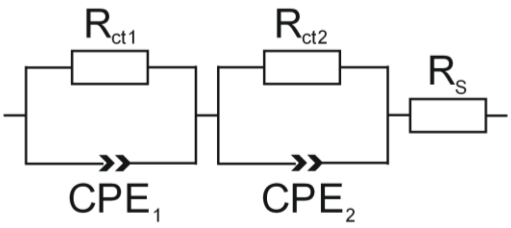

Figure S8. Equivalent electrical circuit used for EIS data fitting

Table S1. Fitting parameters for obtained EIS spectra in 5mM $K_3[Fe(CN)_6]$ + 0.1M KCl solution. The offset voltage was equal to formal potential of the redox couple $[Fe(CN)_6]^{3-/4-}$ with a 10 mV voltage amplitude.

	C-electrode	CNT	CB	Ag	Ag Janus	SiO_2
**R_s, kOhm**	1.10	1.15	1.15	1.11	1.18	1.4
**R_{ct1}, kOhm**	14.9	0.18	0.5	5.8	5.4	4.7
**Y_1, µS·s^a**	1.33	14.4	5.28	3.4	1.75	2.6
**a_1**	0.936	0.727	0.838	0.883	0.885	0.942
**R_{ct2}, kOhm**		3.6	13			3.8
**Y_2, µS·s^a**		1200	1880			36
**a_2**		0.729	0.424			0.730
**Goodness of Fit**	257E-6	6.8E-06	118E-6	690E-06	837E-6	82E-06

Table S2. Values for calculation of active surface area, charge transfer resistance R_{ct} and heterogeneous rate constant k_0 of electrodes for SPE and differently modified SPEs by various carriers. The active surface area values are obtained from CV data, measured in in 5 mM $K_3[Fe(CN)_6]$ + 0.1M KCl solution by fitting the reduction peak value by Randles-Sevcik equation

	slope, $\mu A/mV^{1/2}$	slope, $A/V^{1/2}$	C, $\mu mol/cm^3$	A, cm^2	Rct, Ohm	k_0, $cm \cdot s^{-1}$
**SPE**	1.9	0.000059	5	0.0163	14900	0.000219
**CNT**	8.5	0.00027	5	0.0747	180	0.003956
**CB**	12.23	0.00039	5	0.0256	493	0.001007
**Ag**	4.5	0.000143	5	0.0396	5800	0.000232
**Ag Janus**	5.6	0.000177	5	0.0491	5400	0.000201
**SiO_2**	4.6	0.000146	5	0.0406	8500	0.000154

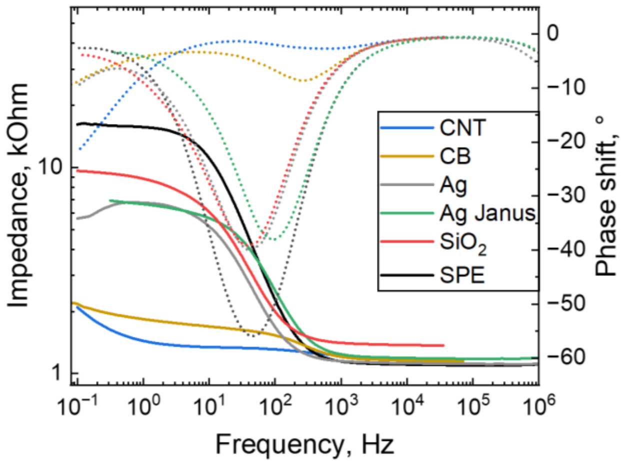

Figure S9. Bode plot of SPE and SPE modified electrodes obtained in 5 mM $K_3[Fe(CN)_6]$ and 0.1 M KCl solution at formal potential offset with 10 mV amplitude.

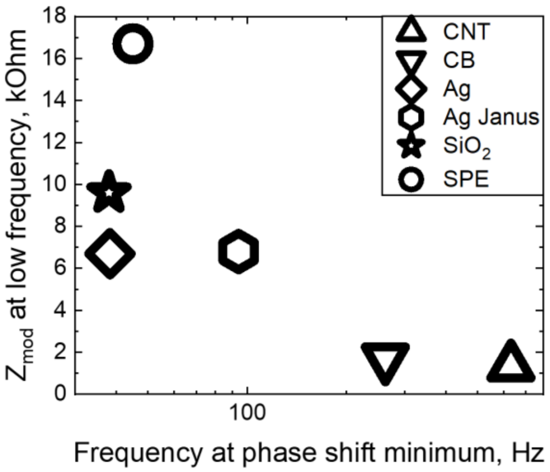

Figure S10. Z_{mod} at low frequency vs frequency at phase shift minimum obtained from Bode plot of EIS measurements in 5 mM $K_3[Fe(CN)_6]$ and 0.1 M KCl solution at formal potential offset with 10 mV amplitude.

Bioelectrocatalysis

SiO_2 – PDMAEMA carrier[1]

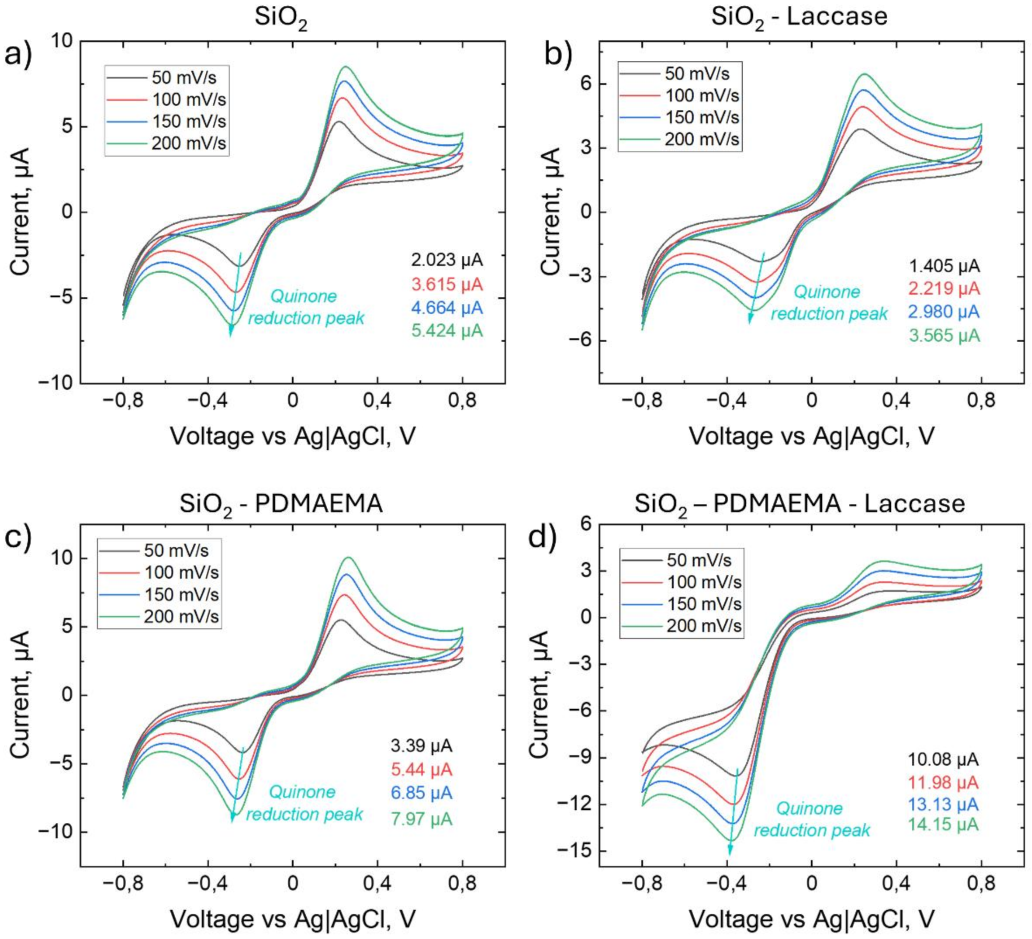

Figure S11. CVA curves for SiO_2 carriers of different modifications-based electrode measured in 0.1 mM hydroquinone solution in 0.1M pH=4 acetate buffer at different scan rates: a) pristine SiO_2, b) SiO_2 – Laccase; c) SiO_2 – PDMAEMA; d) SiO_2 – PDMAEMA - Laccase[1]

Table S3. Values for calculation of active surface area of electrodes for SiO_2 particles of different modification[1]

	slope, $\mu A/mV^{1/2}$	slope, $A/V^{1/2}$	constant, $C \cdot mol^{-1} \cdot V^{-1/2}$	n	D, cm^2/s	C, mol/cm^3	A, cm^2
**SiO_2**	0,48	1,53E-05	2,69E+05	2	7,3E-06	1,0E-07	7,43E-02
**SiO_2- Laccase**	0,31	9,76E-06	2,69E+05	2	7,3E-06	1,0E-07	4,75E-02
**SiO_2 - PDMAEMA**	0,65	2,05E-05	2,69E+05	2	7,3E-06	1,0E-07	9,97E-02
**SiO_2 - PDMAEMA-Laccase**	0,57	1,81E-05	2,69E+05	2	7,3E-06	1,0E-07	8,81E-02

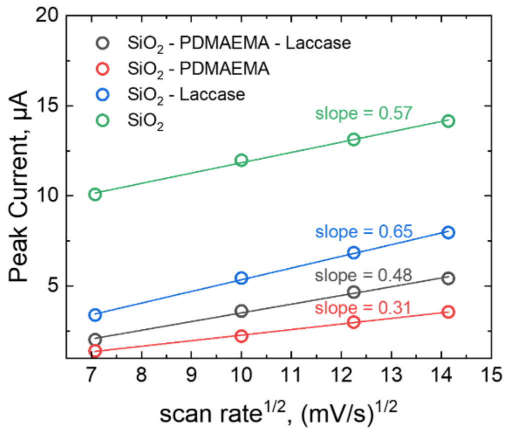

Figure S12. Reduction peak value vs square route of scan rate and its linear fit for SiO_2 carriers of different modifications-based electrode measured in 0.1 mM hydroquinone solution in 0.1M pH=4 acetate buffer[1].

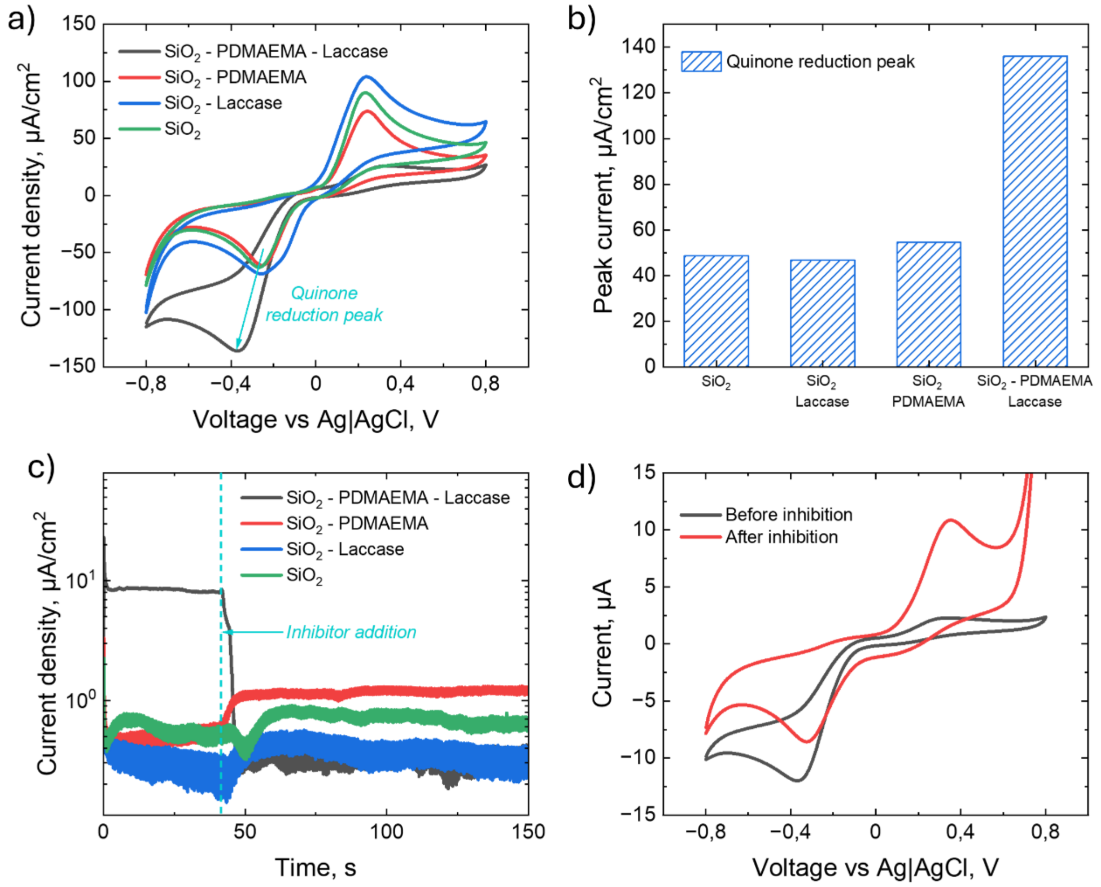

Figure S13. a) CV of differently modified electrodes measured in 0.1 mM hydroquinone solution in 0.1M pH=4 acetate buffer at 100 mV/s; b) Quinone reduction peak values taken from CV results; c) chronoamperometry measurements at constant stirring with addition of NaN_3 solution as inhibitor; d) CV of SiO_2 - PDMAEMA – Laccase electrode before and after inhibition (measured at 100 mV/s)[1]

CNT – PDMAEMA carrier

Carrier	Brush thickness (TGA), nm	Grafting density, chain/nm^2	Polymer M_n, kDa	Polymer M_w, kDa	Activity U/mg of particles	Activity U/mg of polymer	Activity U/mg of enzyme
**CNT – PDMAEMA - Laccase**	2.5	0.12	17.2	43.7	3.6	7.9	--

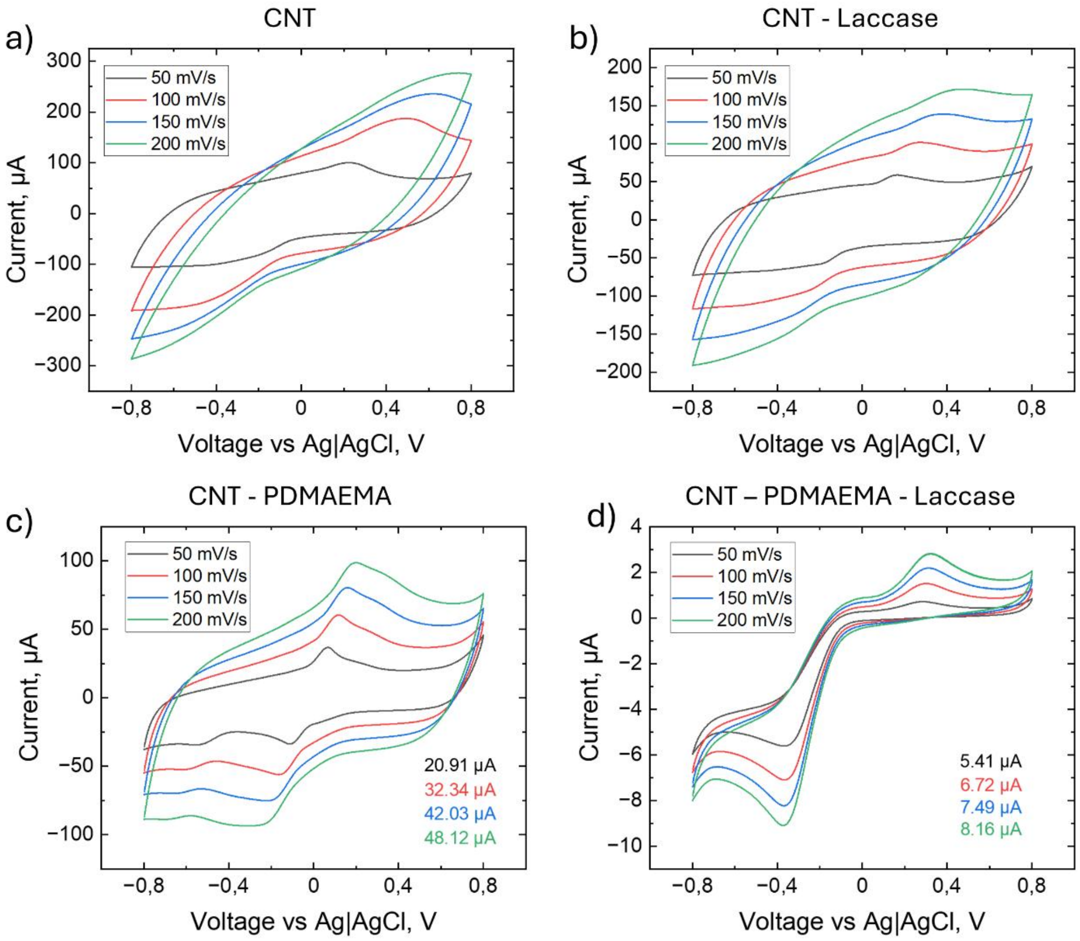

Figure S14. CV curves for CNT carriers of different modifications-based electrode measured in 0.1 mM hydroquinone solution in 0.1M pH=4 acetate buffer at different scan rates: a) pristine CNT, b) CNT – Laccase; c) CNT – PDMAEMA; d) CNT – PDMAEMA – Laccase

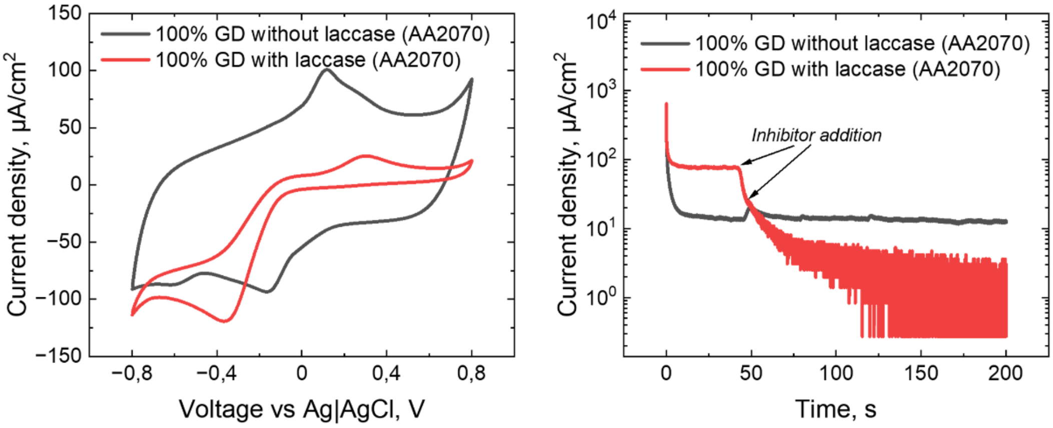

Figure S15. (i) CV in 0.1mM hydroquinone solution in 0.1M pH=4 acetate buffer and (ii) inhibition by 0.1M NaN_3 solution of CNT-PDMAEMA-Laccase based sensor with grafting density 100%.

CB – PDMAEMA carrier

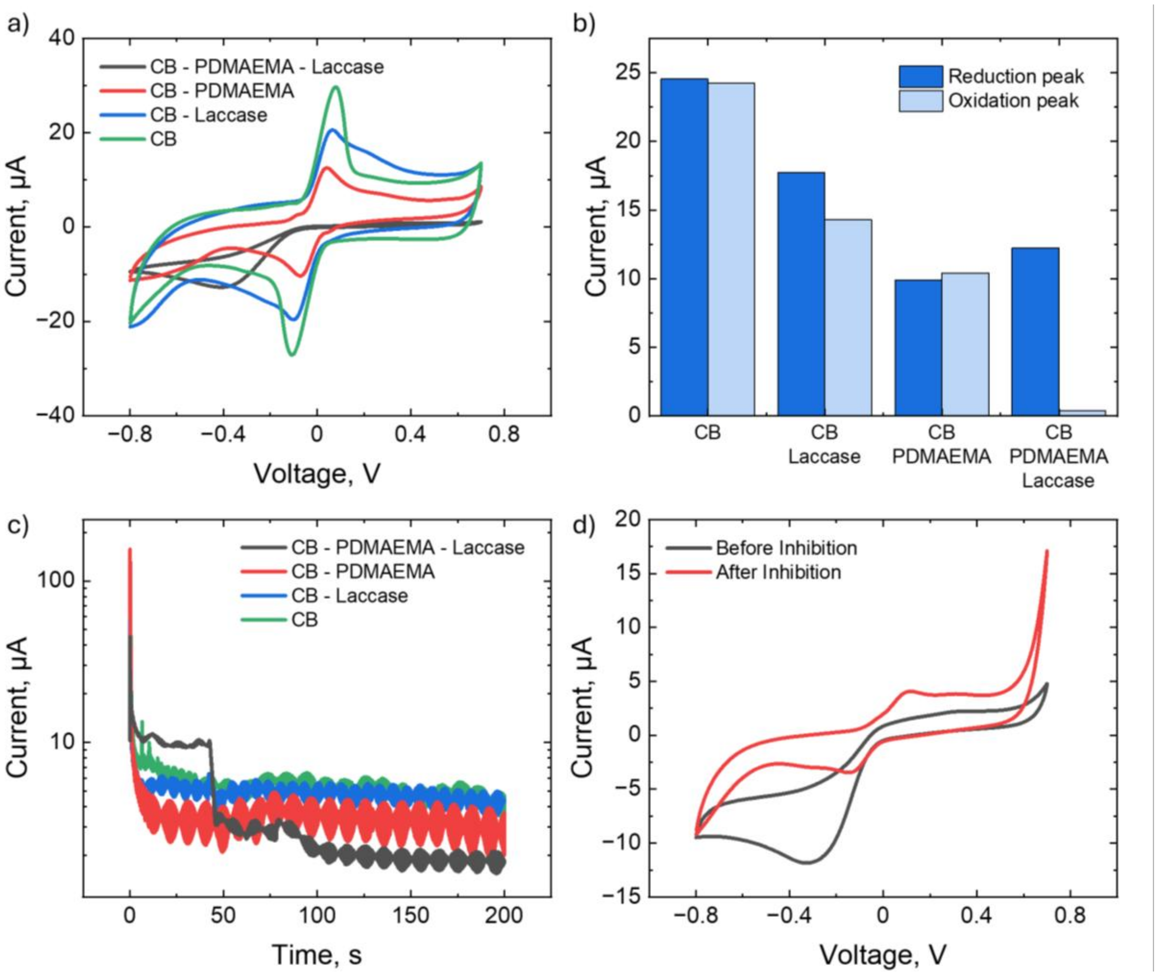

Figure S16. a) CV of differently modified electrodes measured in 0.1 mM hydroquinone solution in 0.1M pH=4 acetate buffer at 100 mV/s; b) Quinone reduction an oxidation peak values taken from CV results; c) chronoamperometry measurements at constant stirring with

addition of NaN_3 solution as inhibitor; d) CVA of CB - PDMAEMA – Laccase electrode before and after inhibition (measured at 100 mV/s)

Ag-Janus – PDMAEMA carrier

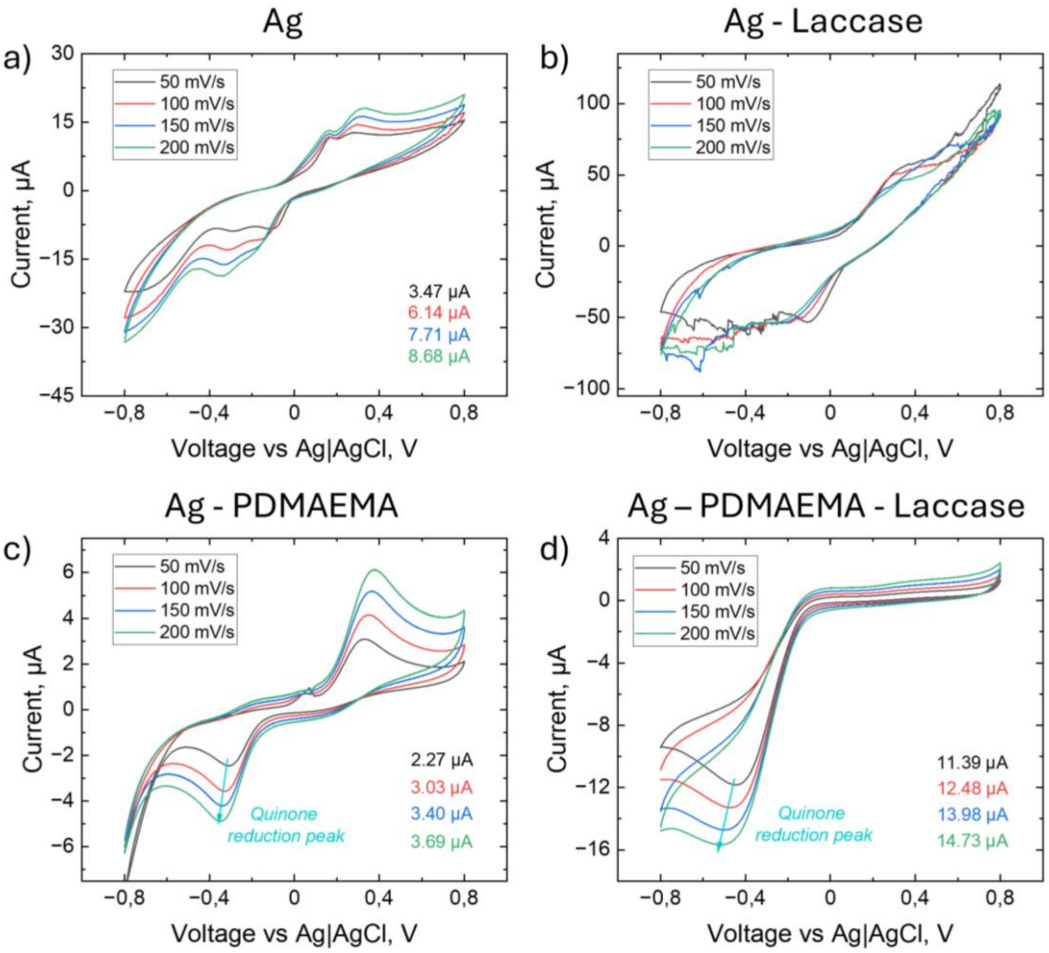

Figure S17. CV curves for Ag-Janus carriers of different modifications-based electrode measured in 0.1 mM hydroquinone solution in 0.1M pH=4 acetate buffer at different scan rates: a) pristine Ag-Janus, b) Ag-Janus – Laccase; c) Ag-Janus – PDMAEMA; d) Ag-Janus – PDMAEMA - Laccase

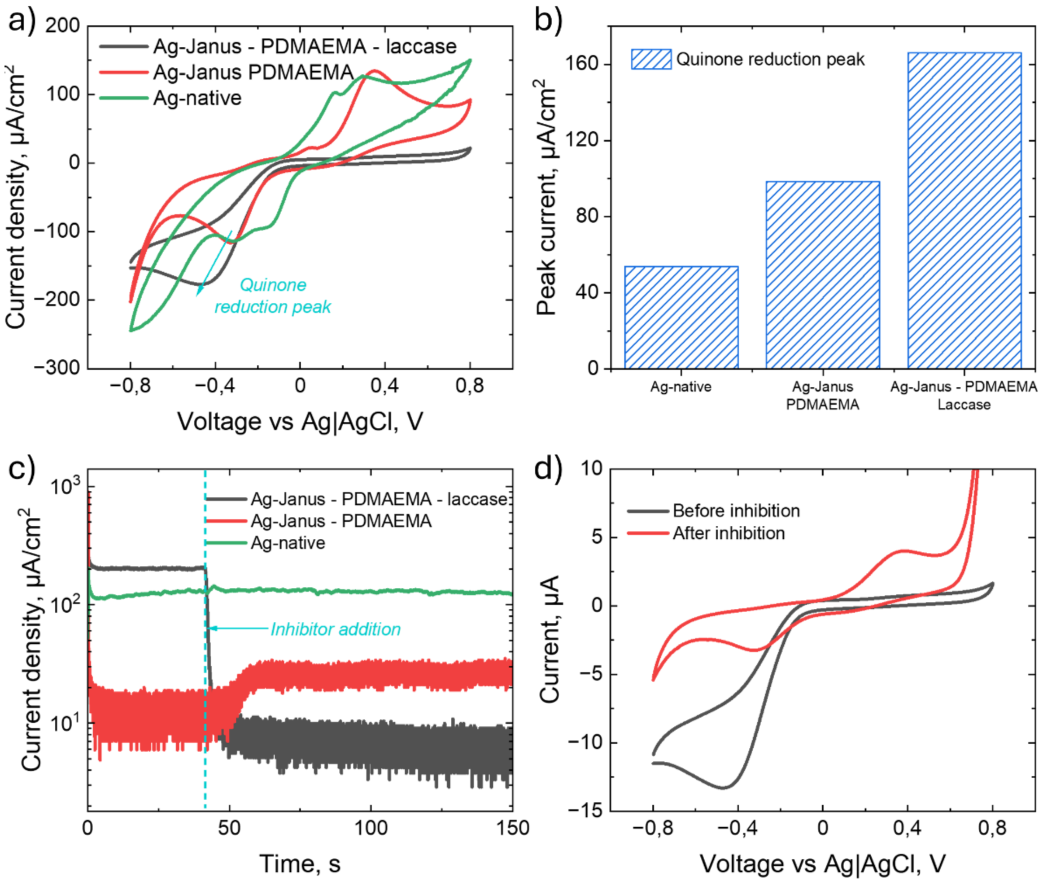

Figure S18. a) CV of differently modified electrodes measured in 0.1 mM hydroquinone solution in 0.1M pH=4 acetate buffer at 100 mV/s; b) Quinone reduction peak values taken from CV results; c) chronoamperometry measurements at constant stirring with addition of NaN_3 solution as inhibitor; d) CV of Ag-Janus - PDMAEMA – Laccase electrode before and after inhibition (measured at 100 mV/s)

Ag – PDMAEMA carrier

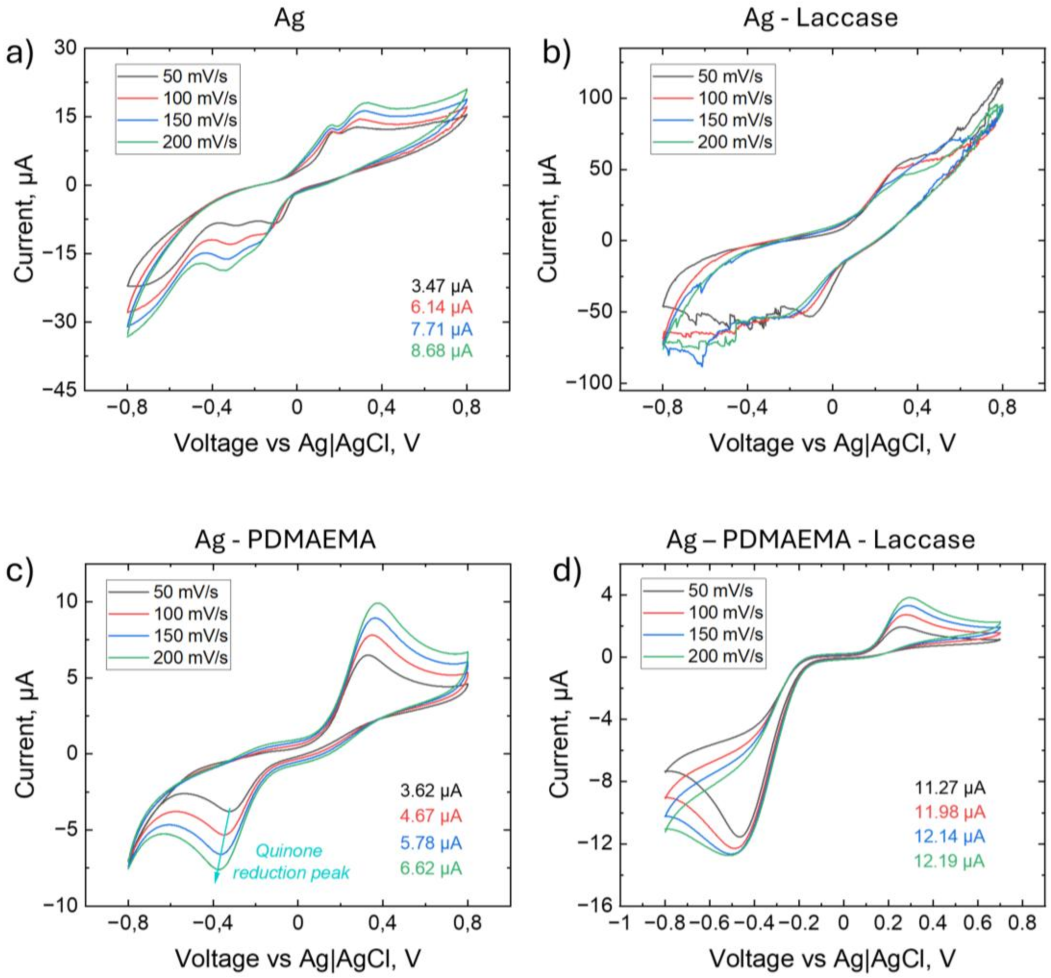

Figure S19. CV curves for SiO_2 carriers of different modifications-based electrode measured in 0.1 mM hydroquinone solution in 0.1M pH=4 acetate buffer at different scan rates: a) pristine Ag, b) Ag – Laccase; c) Ag – PDMAEMA; d) Ag – PDMAEMA - Laccase

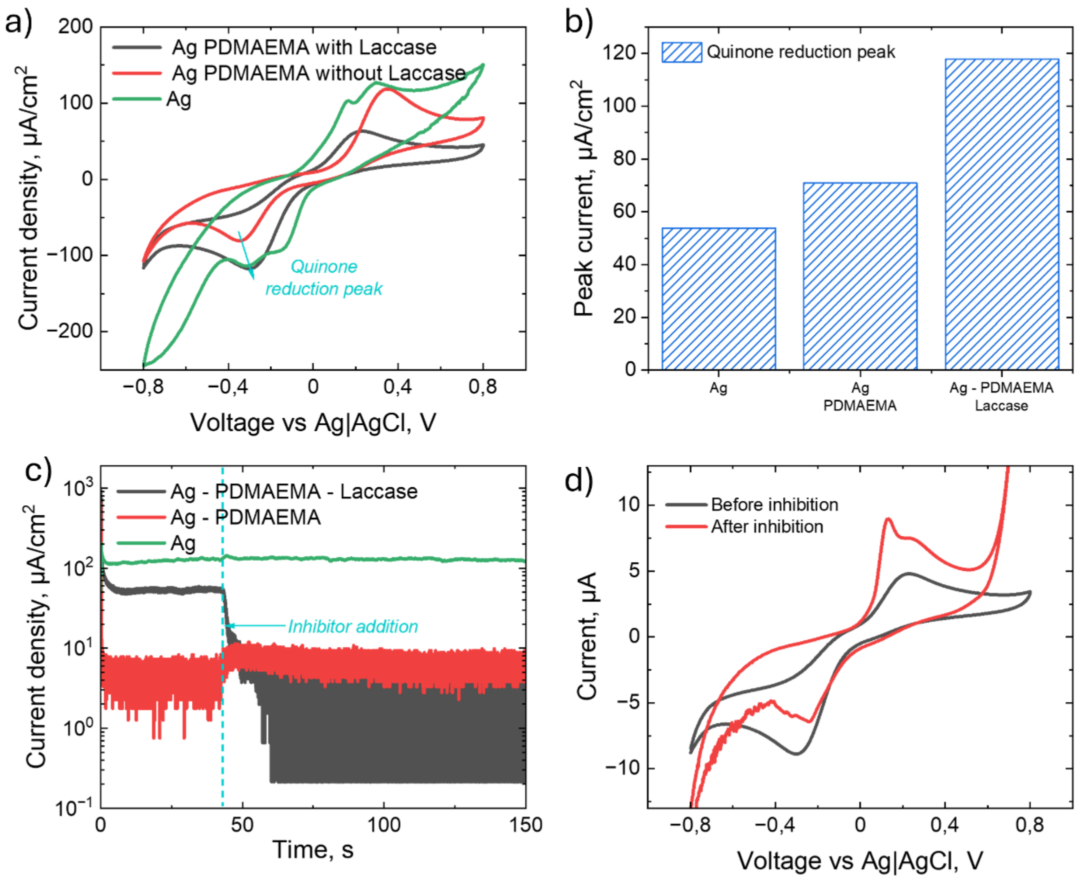

Figure S20. a) CV of differently modified electrodes measured in 0.1 mM hydroquinone solution in 0.1M pH=4 acetate buffer at 100 mV/s; b) Quinone reduction peak values taken from CV results; c) chronoamperometry measurements at constant stirring with addition of NaN_3 solution as inhibitor; d) CV of Ag - PDMAEMA – Laccase electrode before and after inhibition (measured at 100 mV/s)

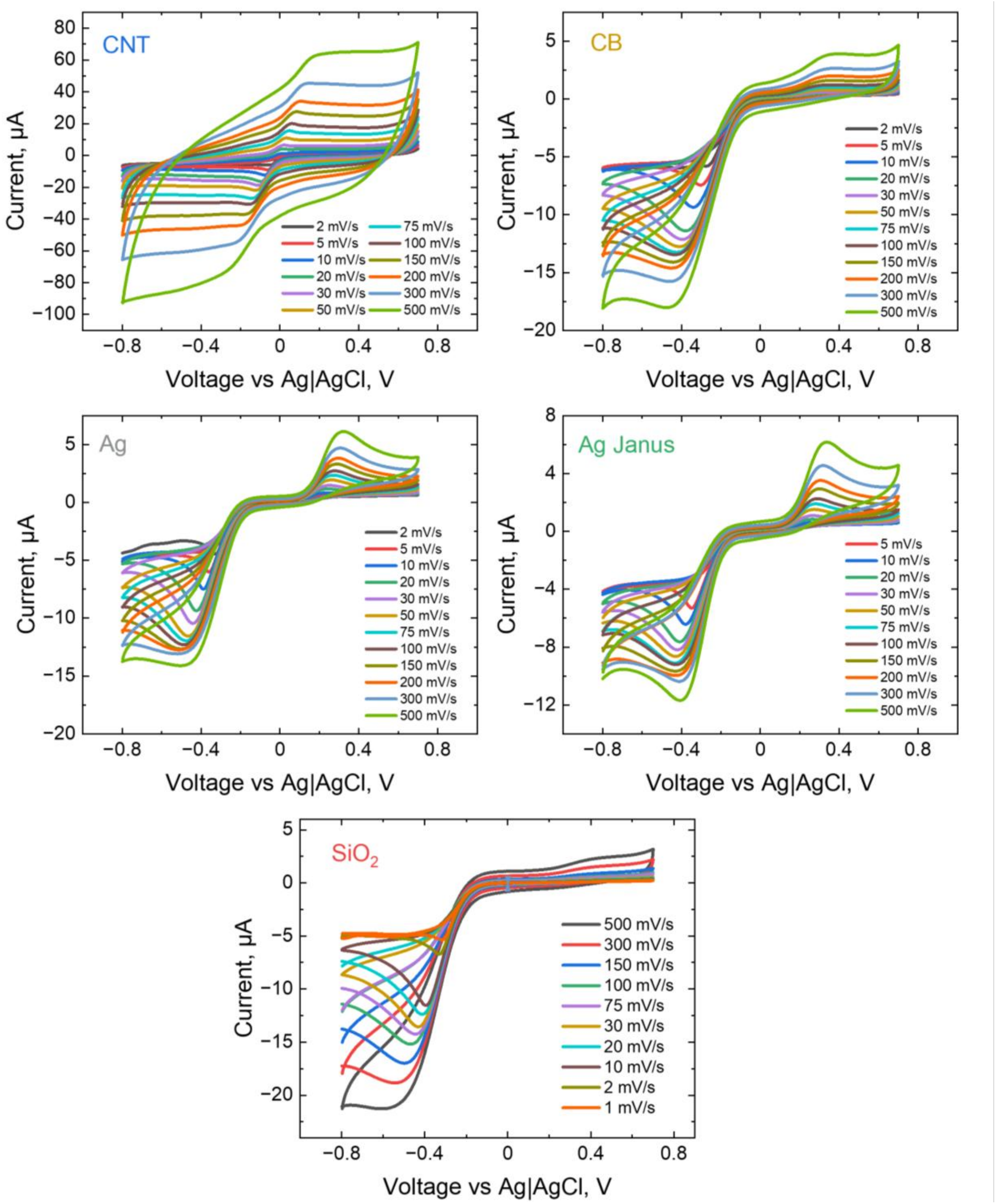

Figure S21. CV of differently modified SPE electrode at various scan rates in 0.1 mM HQ solution in 0.1 M acetic buffer pH=4.

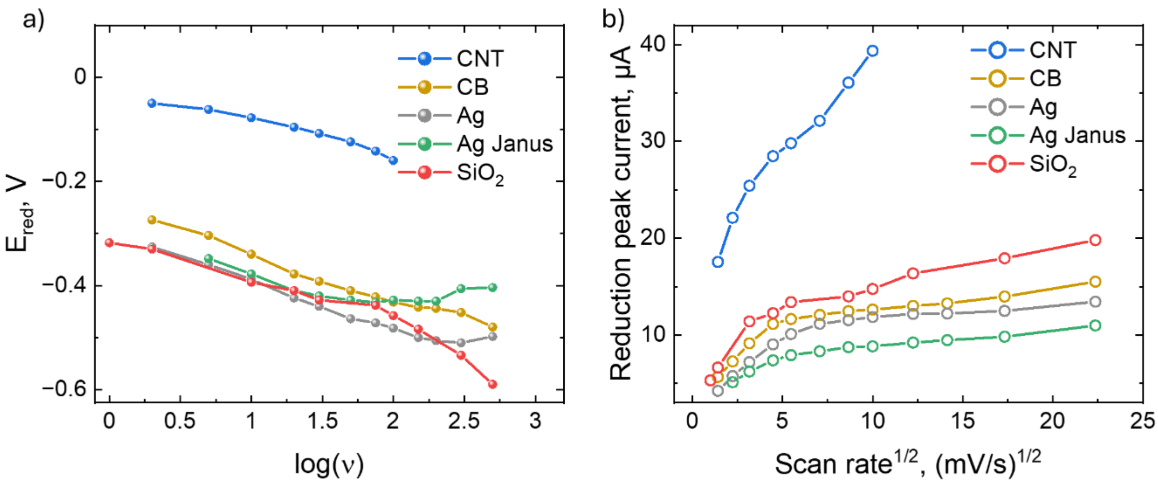

Figure S22. a) Reduction peak of quinone vs log(v) obtained by CV measurements in 0.1mM HQ solution in 0.1 M acetic buffer pH=4 at different scan rate (v), mV/s for differently modified SPEs; b) Quinone reduction peak value vs $v^{1/2}$ obtained during the same measurements

Sensor parameters and calibration

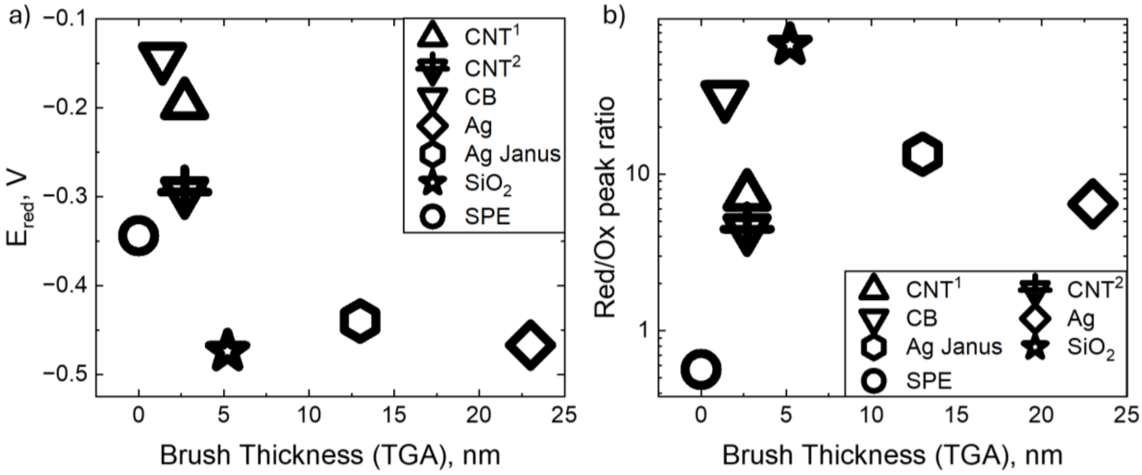

Figure S23. a) reduction peak potential and b) Red/Ox peak ratio vs. brush thickness (TGA). The values obtained from CV measurements of electrodes in 0.1M HQ in 0.1M pH=4 acetate buffer solution at 50 mV/s.

Calibration: chronoamperometry

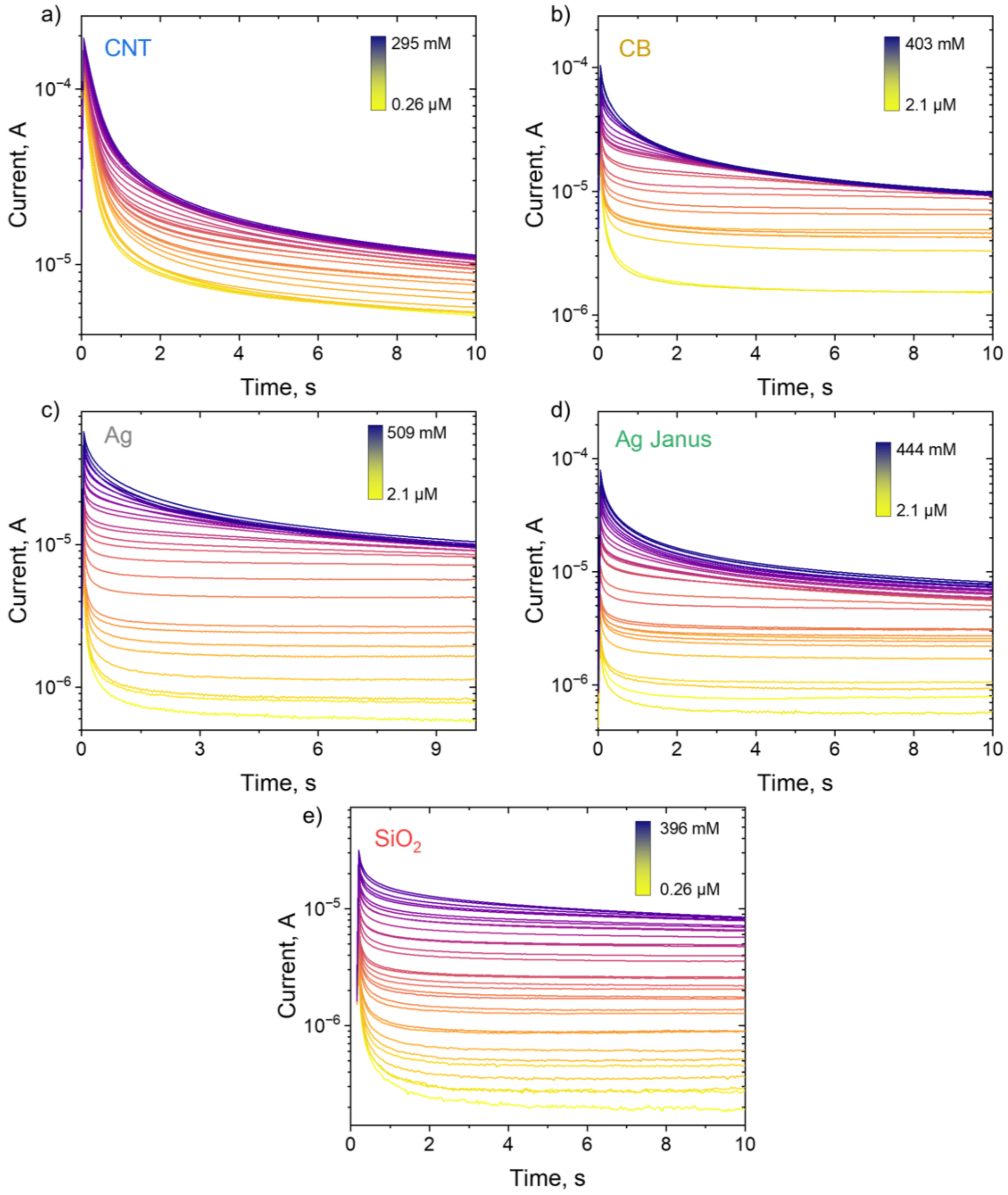

Figure S24. Chronoamperometric curves obtained in different concentrations of hydroquinone solution in 0.1M pH=4 acetate buffer for differently modified SPEs. Data for SiO_2 are taken from our previous study[1].

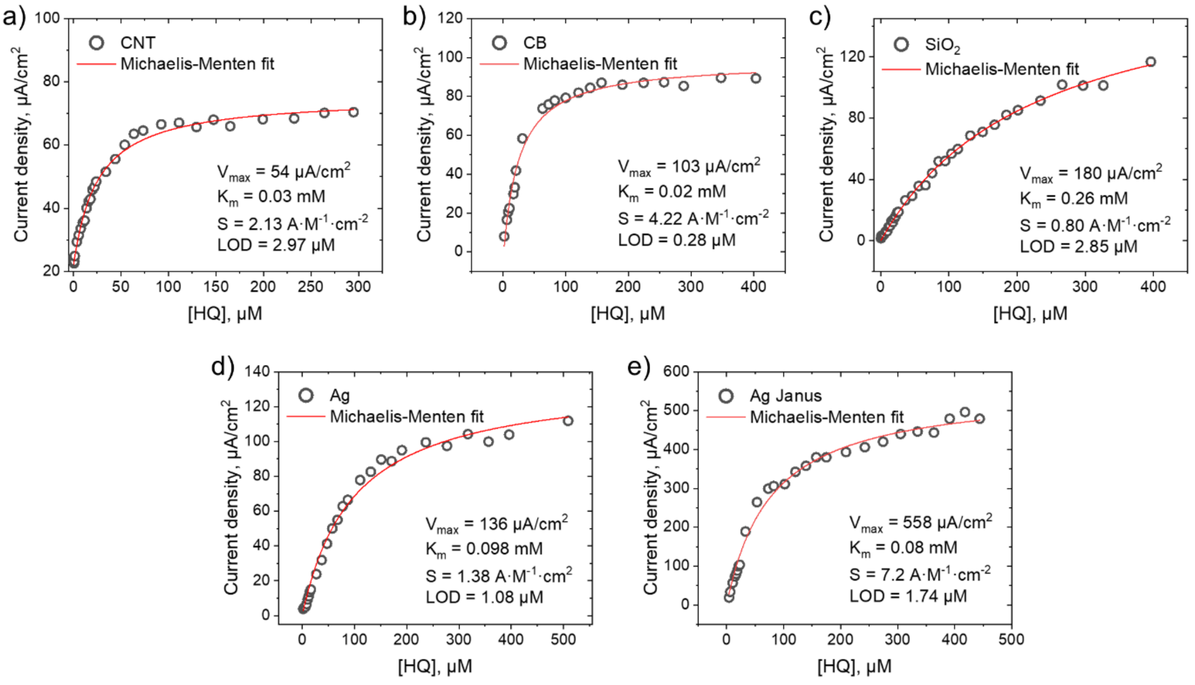

Figure S25. Calibration curves of a) CNT, b) CB, c) $SiO_{2[1]}$, d) Ag, e) Ag Janus carriers modified SPEs with immobilised laccase obtained by chronoamperometry in HQ solutions in 0,1M pH=4 acetate buffer at potential E=-0.6 V vs Ag|AgCl electrode. The points correspond to current at t=2s. The calibration curve for all carriers follows Michaelis-Menten equation.

$i = i_0 + \frac{i_{max}\cdot[HQ]}{K_m+[HQ]}$, where [S] – substrate concentration, imax – maximum current density at saturating substrate concentration at a given enzymes concentration, K_m – Michaelis constant. For CNT carrier an additional term was used in the equation because of high background current due to capacitance like behaviour of CNTs. For sensor comparison, the current was normalized on active surface area as well.

The sensor parameters are demonstrated in Table X. The sensitivity of sensor correlates with results of CV: the higher the peak current, the higher the sensitivity. Thus, the sensitivity 7.2 $A\cdot M^{-1}\cdot cm^{-2}$ was achieved for Ag Janus carrier. The least sensitive was SiO_2 based sensor with $S = 0.8\ A\cdot M^{-1}\cdot cm^{-2}$. The LOD correlates with k0 and Michaelis constant values. Thus, CB with one of the highest rate constants and lowest K_m = 20 μM demonstrated the best performance with LOD = 0.28 μM. Though, CNT has the highest k_0 value and comparable with CB Michaelis constant, the capacitance impact to the current leads to high background current, increasing the LOD.

Table S4. Parameters of sensors obtained via calibration using chronoamperometry technique at E = -0.6 V at various HQ concentration in 0.1M pH = 4 acetic buffer

	CNT	CB	Ag	Ag Janus	SiO_2
**S, $A{\cdot}M^{-1}{\cdot}cm^{-2}$**	2.13	4.22	1.38	7.2	0.8
**K_m, mM**	0.03	0.02	0.098	0.08	0.26
**LOD, µM**	2.97	0.28	1.08	1.74	2.85
**Detection range, µM**	2.97 - 29	0.28 - 47	1.08 - 80	1.74 – 77.5	2.85 - 85

Calibration: DPV and NPV

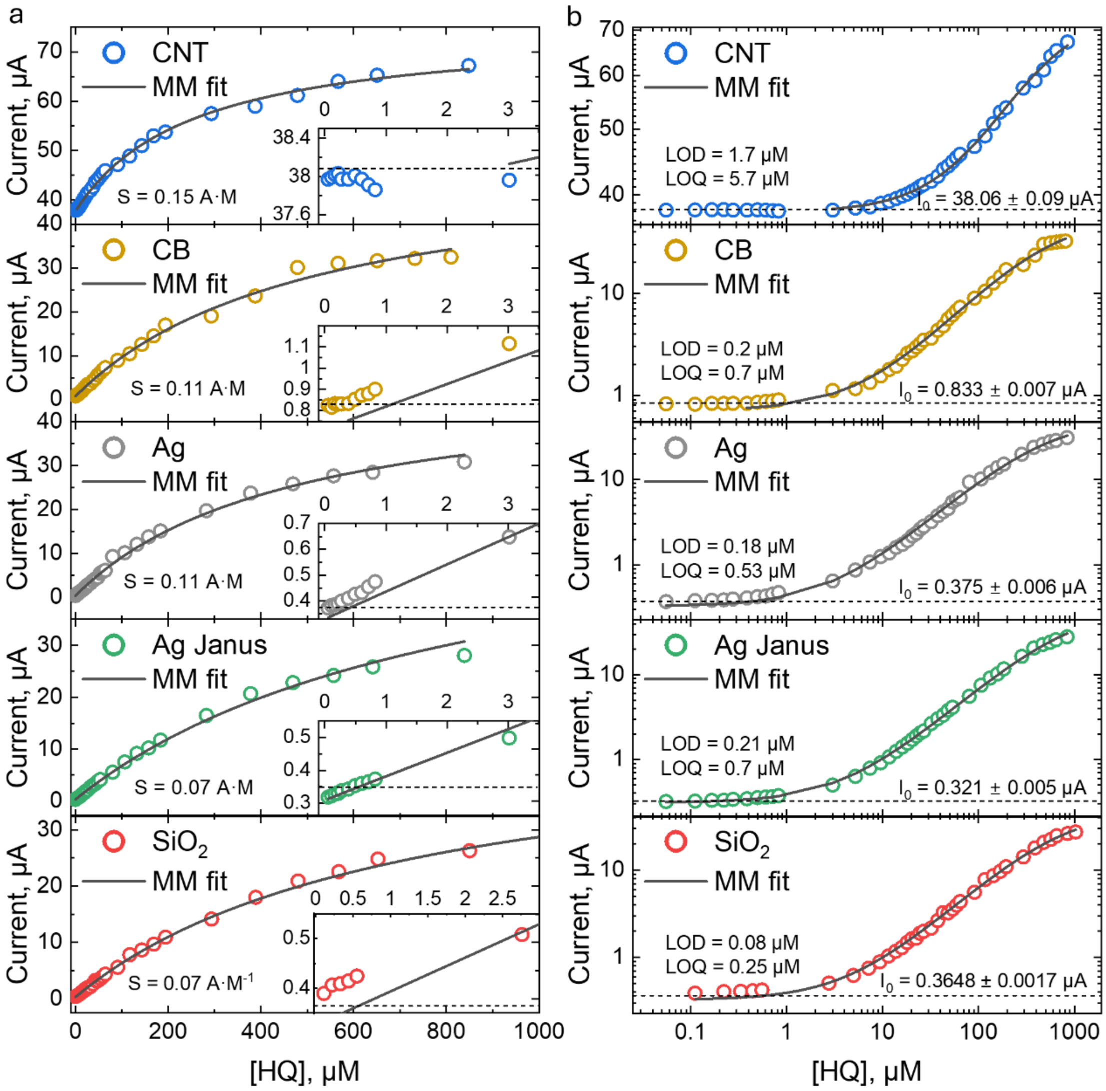

Figure S26. DPV calibration for HQ solutions of different concentrations in 0.1 m pH = 4 acetate buffer plotted in (a) lin-lin and (b) log-log scale. The DPV was performed with 0.1 s pulse time, 1s sample period, 10 mV step size, and 200 mV pulse. Dash – line shows themean value of the blank buffer solution with its standard deviation. Data for SiO_2 are taken from our previous study[1]

Table S5. Sensor parameters obtained from calibration curves measured by DPV

	**CNT**	**CB**	**Ag**	**Ag Janus**	**SiO_2[1]**
**S, $A \cdot M^{-1}$**	0.15	0.11	0.11	0.07	0.07
**LOD, µM**	1.7	0.2	0.18	0.21	0.08
**Detection range, µM**	5.7 - 130	0.7 - 260	0.53 - 235	0.7 – 425	0.25 - 350

** Upper limit of detection range was determined as 0.5 × K_m, LOD was determined as 3·σ/S, where σ – standard deviation of background current (empty buffer). Lower limit of detection rang was estimated as LOQ = 10·σ/S.*

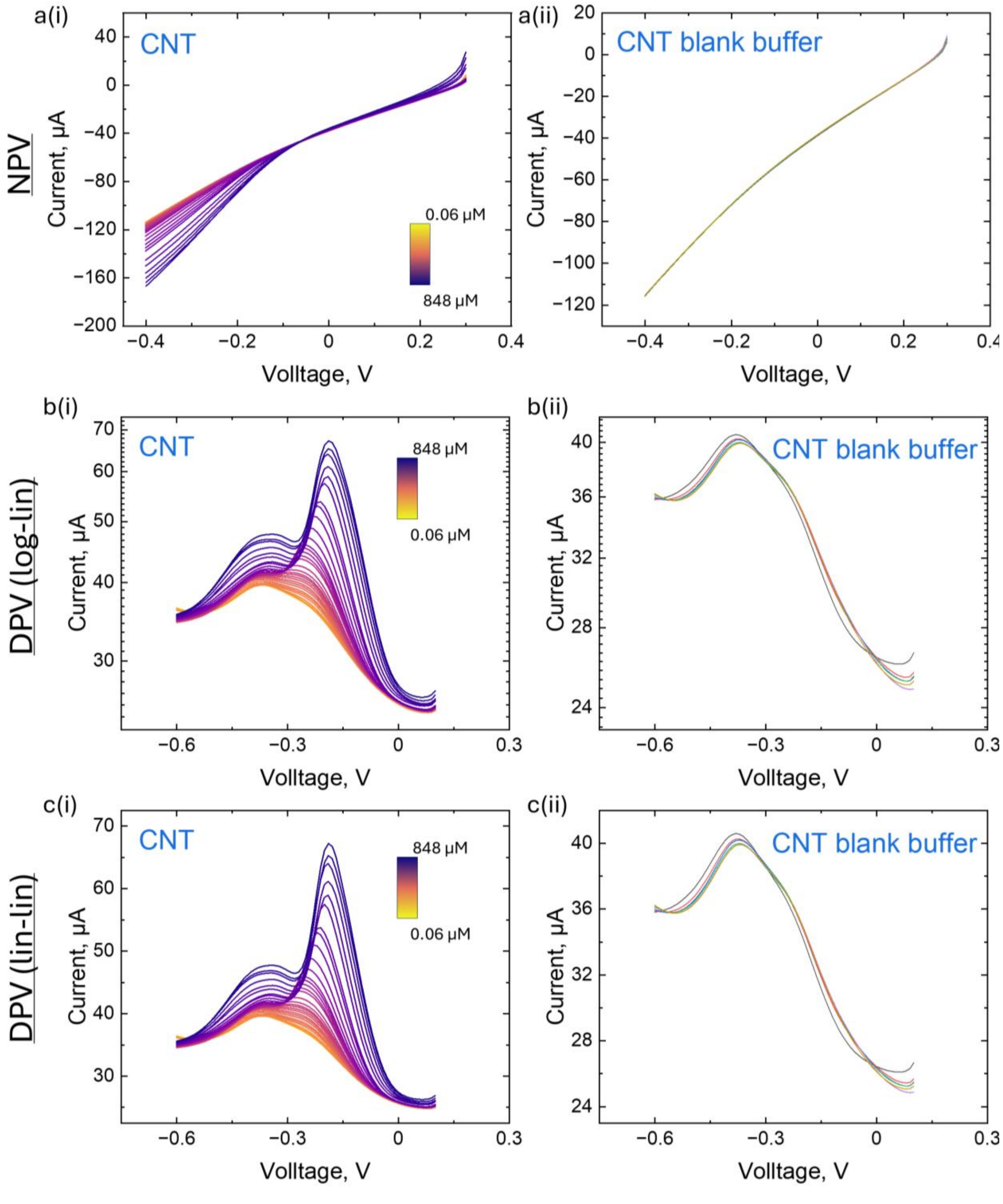

Figure S27. CNT – PDAMAEMA carrier: a) Normal pulse voltammetry (NPV), b) differential pulse voltammetry (DPV) in log-lin scale and c) DPV in lin-lin scale for (i) HQ solutions of different concentrations in 0.1M pH = 4 acetate buffer and (ii) for pure acetate buffer. The NPV was performed with 0.1s pulse time, 1s sample period and 10 mV step size. The DPV was performed with 0.1s pulse time, 1s sample period, 10mV step size and 200 mV pulse.

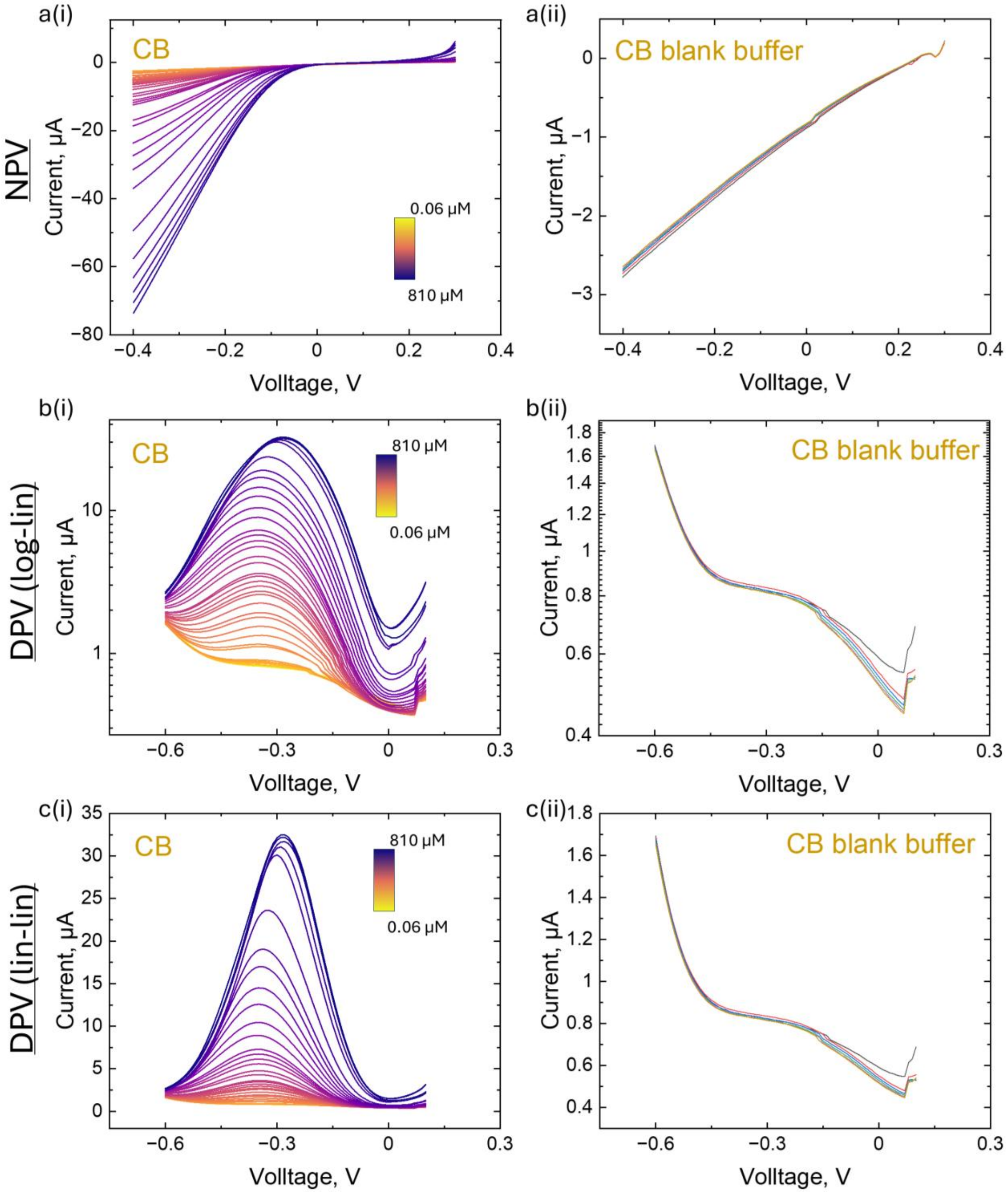

Figure S28. CB – PDAMAEMA carrier: a) Normal pulse voltammetry (NPV), b) differential pulse voltammetry (DPV) in log-lin scale and c) DPV in lin-lin scale for (i) HQ solutions of different concentrations in 0.1M pH = 4 acetate buffer and (ii) for pure acetate buffer. The NPV was performed with 0.1s pulse time, 1s sample period and 10 mV step size. The DPV was performed with 0.1s pulse time, 1s sample period, 10mV step size and 200 mV pulse.

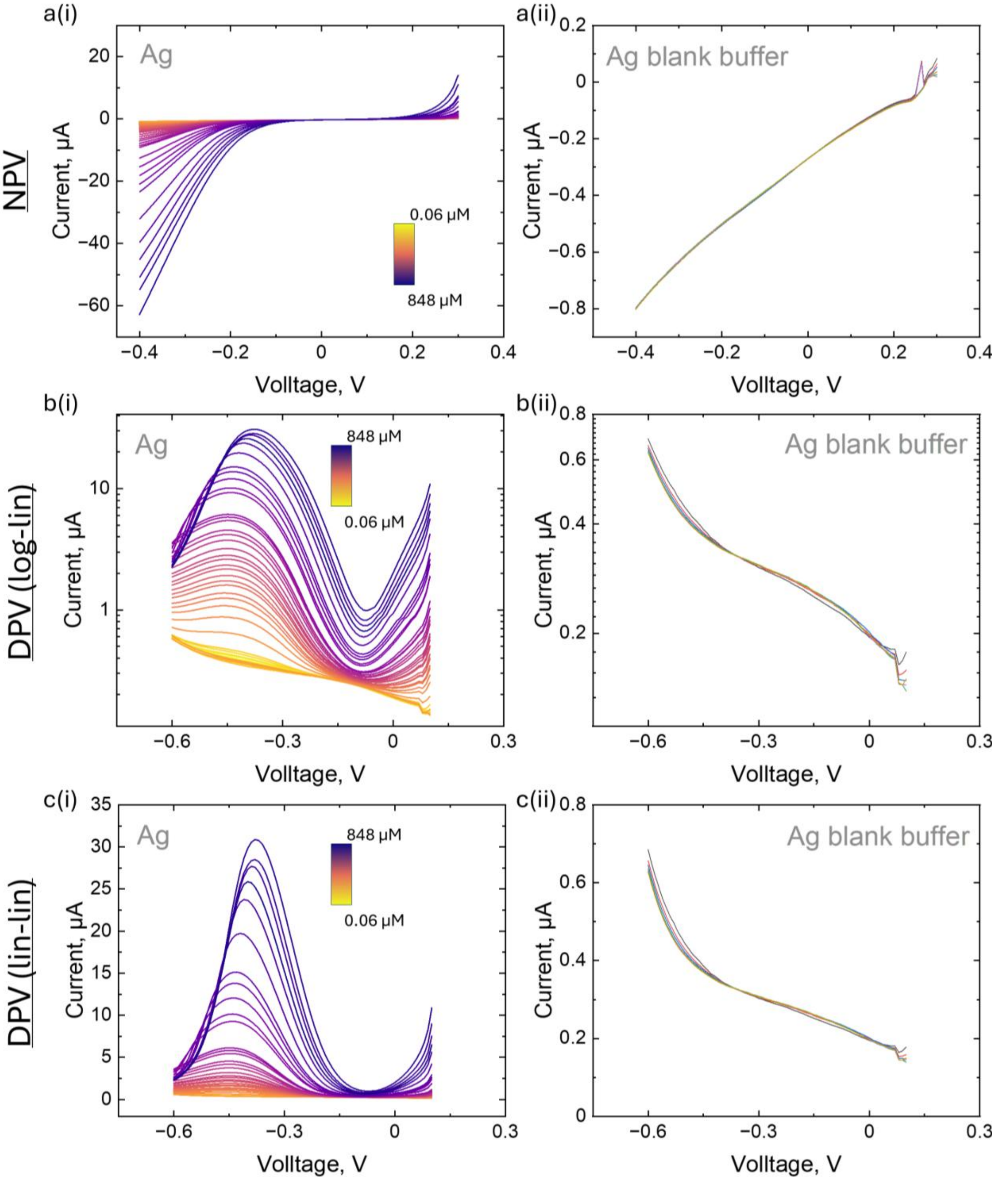

Figure S29. Ag – PDAMAEMA carrier: a) Normal pulse voltammetry (NPV), b) differential pulse voltammetry (DPV) in log-lin scale and c) DPV in lin-lin scale for (i) HQ solutions of different concentrations in 0.1M pH = 4 acetate buffer and (ii) for pure acetate buffer. The NPV was performed with 0.1s pulse time, 1s sample period and 10 mV step size. The DPV was performed with 0.1s pulse time, 1s sample period, 10mV step size and 200 mV pulse.

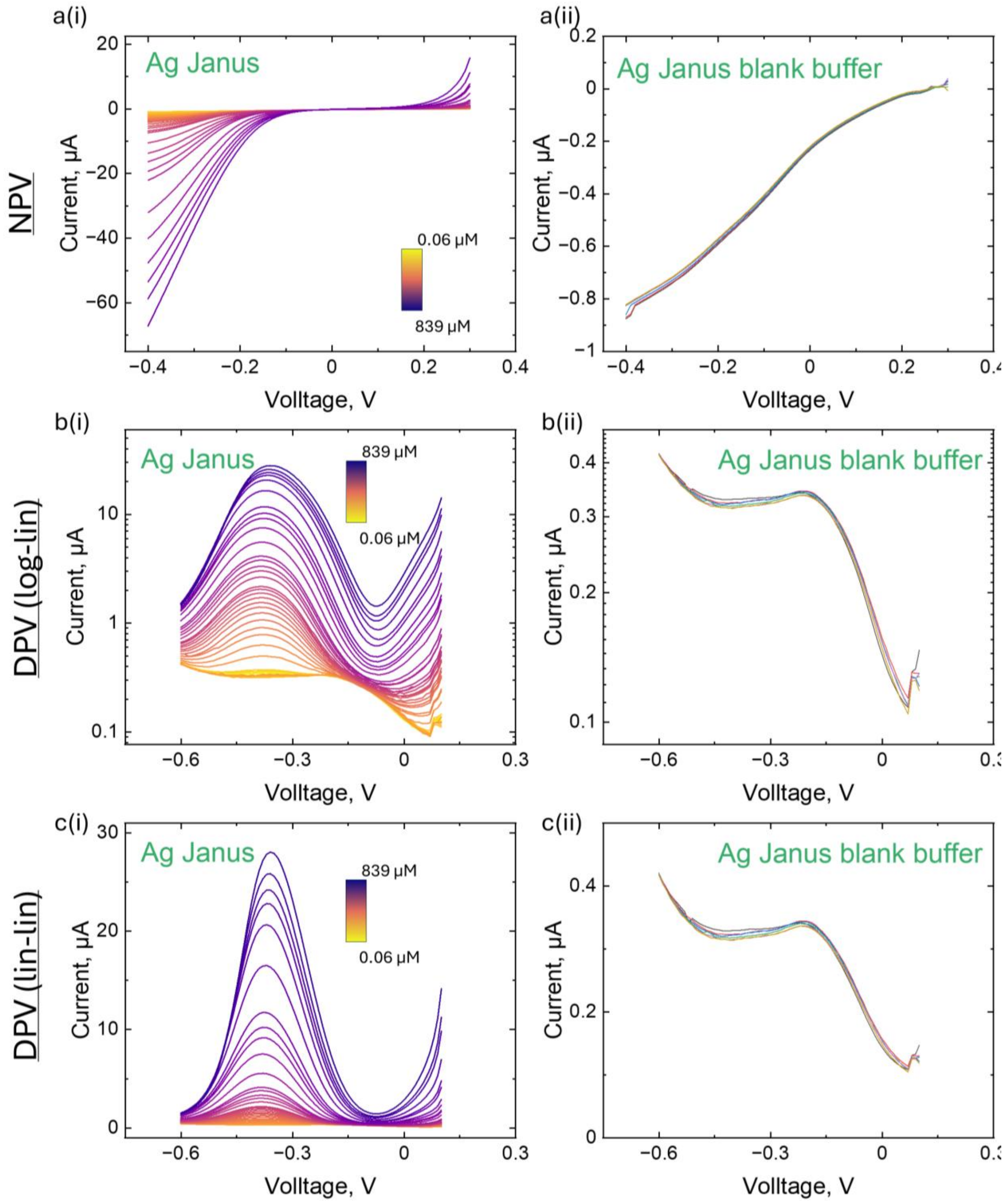

Figure S30. Ag Janus – PDAMAEMA carrier: a) Normal pulse voltammetry (NPV), b) differential pulse voltammetry (DPV) in log-lin scale and c) DPV in lin-lin scale for (i) HQ solutions of different concentrations in 0.1M pH = 4 acetate buffer and (ii) for pure acetate buffer. The NPV was performed with 0.1s pulse time, 1s sample period and 10 mV step size. The DPV was performed with 0.1s pulse time, 1s sample period, 10mV step size and 200 mV pulse.

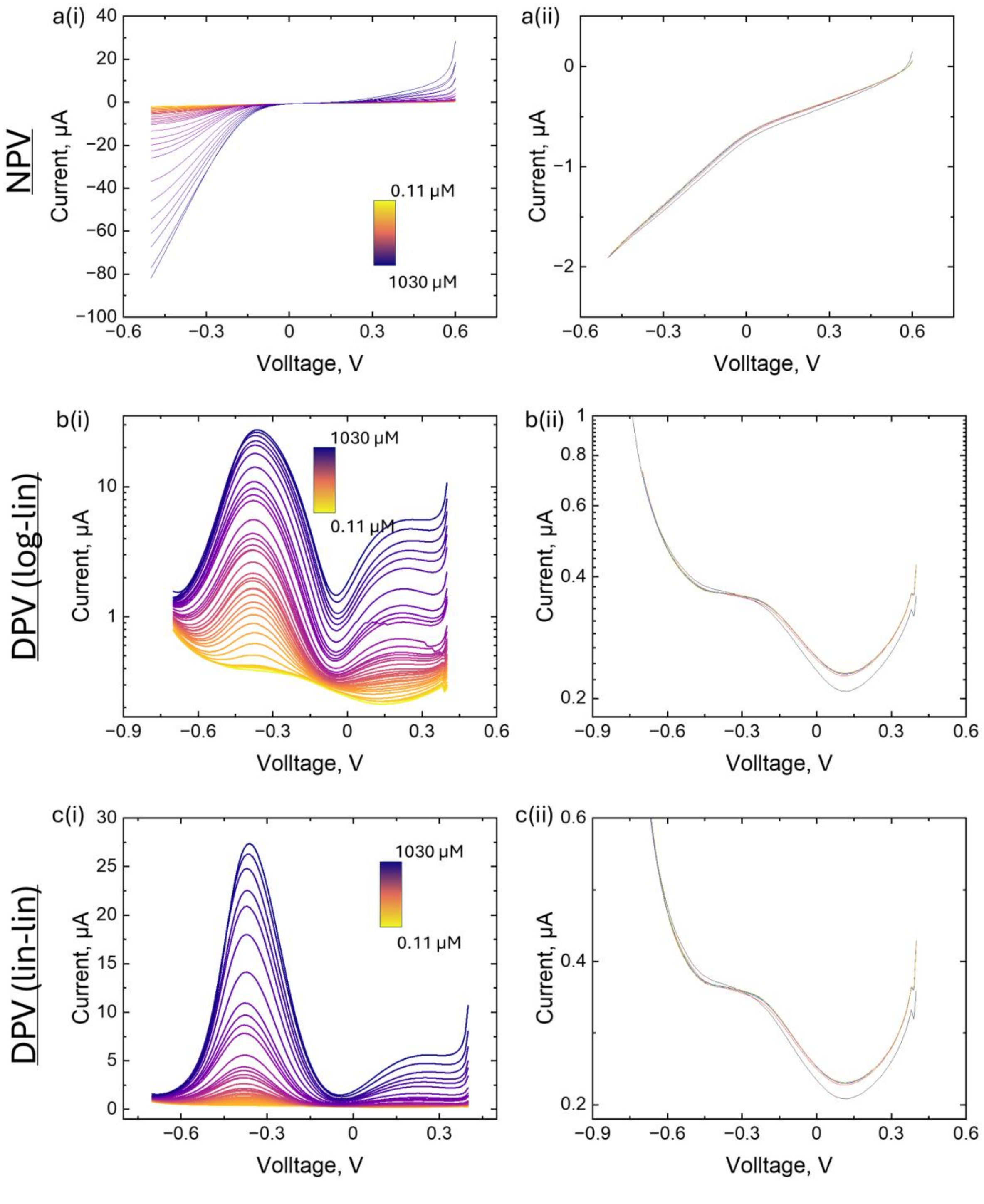

Figure S31. SiO_2 – PDAMAEMA carrier[1]: a) Normal pulse voltammetry (NPV), b) differential pulse voltammetry (DPV) in log-lin scale and c) DPV in lin-lin scale for (i) HQ solutions of different concentrations in 0.1M pH = 4 acetate buffer and (ii) for pure acetate buffer. The NPV was performed with 0.1s pulse time, 1s sample period and 10 mV step size. The DPV was performed with 0.1s pulse time, 1s sample period, 10mV step size and 200 mV pulse.

Capacitive vs Faradaic current

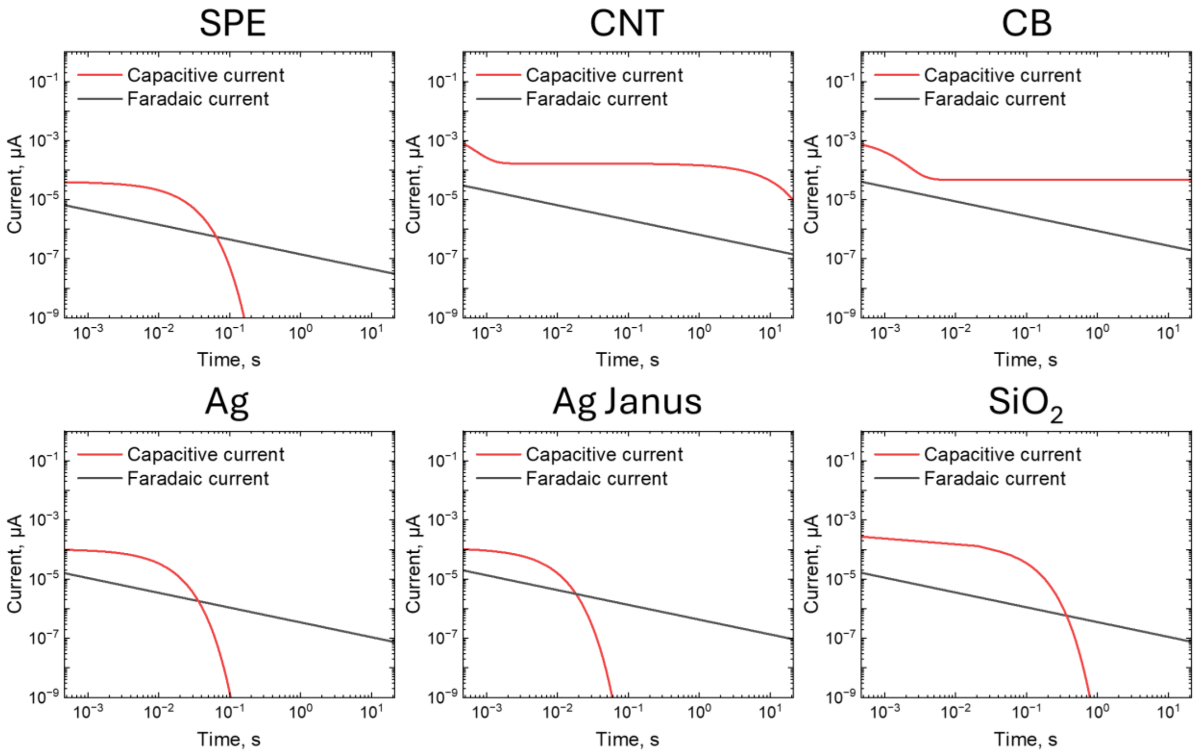

Figure S32. The capacitive and Faradaic current simulations for bare and differently modified SPE. The Faradaic current was calculated according to the Cotrell equation $i_f = \frac{nFAC\sqrt{D}}{\sqrt{\pi t}}$, where n – number of electrons, F – Faradaic constant C·mol^{-1}, A – active surface area m^2, C – concentration of analyte mol·m^{-3}, D – diffusion coefficient m^2·s^{-1} (2·10^{-10} m^2·s^{-1} for hydroquinone, t – time. The active surface area was taken from Table S1. Concentration of analyte was taken as bulk concentration – 0.1 mM (however, the local concentration of quinone can be higher than in bulk). The capacitive current was estimated as $i_c = \frac{E}{R}e^{-\frac{t}{RC}}$, where E – applied potential, *C* – capacitance and R – corresponding resistance. If the EIS results were fitted with two R-CPE elements, additive function of 2 exponents were used. The corresponding capacitance C was recalculated according to the equation $C = (Y \cdot R^{1-a})^{1/a}$, where *Y* – CPE coefficient, *a* – CPE exponent.

Comparison to other studies

The data of other studies replicates the data in our previous research[1]

Sensitivity, A·M^{-1}	Lower limit of detection range, μM	Lowest measured concentration, μM	Upper limit of detection range, μM	Highest measured concentration, μM	LOD, μM	Ref.
0.111	1.6		409.6		0.3	[2]
1.12	2.9	2.9	22	22	0.21	[3]
0.0246	1	1	89.8	89.8	3.65	[4]
0.03639	0.4	0.4	337.2	337.2	2.94	[5]
0.0029	4	4	130	130	1.5	[6]

0.00253	3	3	15	140	0.89	[7]
0.0029	3	3	15	140	0.91	[7]
0.0284	0.9	3	20	140	0.25	[7]
0.009	1.1	1.1	130	130	1.071	[8]
0.05	1		10		0.58	[9]
0.3	0.1	0.1	0.8	0.8	0.035	[10]
0.01416	3	0.1	2000	5000	2	[11]
28.5	0.5	0.5	2.06	2.06	0.0095	[12]
0.2118	0.1	0.015	137	140	0.015	[13]
0.03964	0.03	0.03	172.98	172.98	0.01	[14]
0.009	0.53	0.53	860	860	0.42	[15]
	10		90		0.10443	[16]
0.55	1	1	100	100	0.75	[17]
0.143	2	2	160	160	1.5	[18]
0.079	4	4	1000	5000	0.63	[19]
0.0018	6	6	100	100	1	[20]
0.0465	1	1	78	84	0.19	[21]
0.0325	4.5	4.5	4500	4500	1.5	[22]
0.373	1	1	200	200	0.2	[23]
177.99	0.01	0.01	0.7	0.7	0.007	[24]
0.6774	0.5	0.5	300	300	0.012	[25]
0.1	14.3		321		4.3	CNT (NPV)
0.15	5.7		130		1.7	CNT (DPV)
0.14	3.2		446		1	CB (NPV)
0.11	0.7		260		0.2	CB (DPV)
0.17	0.12		484		0.04	Ag (NPV)
0.11	0.53		235		0.18	Ag (DPV)
0.12	0.9		655		0.27	Ag Janus (NPV)
0.07	0.7		425		0.21	Ag Janus (DPV
0.14	0.3		750		0.1	SiO2 (NPV)[1]
0.07	0.25		350		0.08	SiO2 (DPV)[1]

References

[1] P. Milkin *et al.*, "Highly Sensitive Electrochemical Biosensor Based on Hairy Particles with Controllable High Enzyme Loading and Activity," *Advanced Functional Materials,* 2025, doi: 10.1002/adfm.202507589.

[2] S. Đurđić *et al.*, "Laccase Polyphenolic Biosensor Supported on MnO2@GNP Decorated SPCE: Preparation, Characterization, and Analytical Application," *Journal of The Electrochemical Society,* vol. 168, no. 3, p. 037510, 2021/03/16 2021, doi: 10.1149/1945-7111/abeaf2.

[3] D. Brondani, B. de Souza, B. S. Souza, A. Neves, and I. C. Vieira, "PEI-coated gold nanoparticles decorated with laccase: A new platform for direct electrochemistry of enzymes and biosensingapplications," *Biosensors and Bioelectronics,* vol. 42, pp. 242-247, 2013/04/15/ 2013, doi: https://doi.org/10.1016/j.bios.2012.10.087.

[4] J. Yang, D. Li, J. Fu, F. Huang, and Q. Wei, "TiO2-CuCNFs based laccase biosensor for enhanced electrocatalysis in hydroquinone detection," *Journal of Electroanalytical Chemistry,* vol. 766, pp. 16-23, 2016/04/01/ 2016, doi: https://doi.org/10.1016/j.jelechem.2016.01.030.

[5] C. Lou *et al.*, "Laccase immobilized polyaniline/magnetic graphene composite electrode for detecting hydroquinone," *International Journal of Biological Macromolecules,* vol. 149, pp. 1130-1138, 2020/04/15/ 2020, doi: https://doi.org/10.1016/j.ijbiomac.2020.01.248.

[6] I. Zrinski *et al.*, "Evaluation of phenolic antioxidant capacity in beverages based on laccase immobilized on screen-printed carbon electrode modified with graphene nanoplatelets and gold nanoparticles," *Microchemical Journal,* vol. 152, p. 104282, 2020/01/01/ 2020, doi: https://doi.org/10.1016/j.microc.2019.104282.

[7] E. Casero *et al.*, "Laccase biosensors based on different enzyme immobilization strategies for phenolic compounds determination," *Talanta,* vol. 115, pp. 401-408, 2013/10/15/ 2013, doi: https://doi.org/10.1016/j.talanta.2013.05.045.

[8] P. Ibarra-Escutia, J. J. Gómez, C. Calas-Blanchard, J. L. Marty, and M. T. Ramírez-Silva, "Amperometric biosensor based on a high resolution photopolymer deposited onto a screen-printed electrode for phenolic compounds monitoring in tea infusions," *Talanta,* vol. 81, no. 4, pp. 1636-1642, 2010/06/15/ 2010, doi: https://doi.org/10.1016/j.talanta.2010.03.017.

[9] A. Jarosz-Wilkołazka, T. Ruzgas, and L. Gorton, "Amperometric detection of mono- and diphenols at Cerrena unicolor laccase-modified graphite electrode: correlation between sensitivity and substrate structure," *Talanta,* vol. 66, no. 5, pp. 1219-1224, 2005/06/15/ 2005, doi: https://doi.org/10.1016/j.talanta.2005.01.026.

[10] A. I. Yaropolov *et al.*, "An Amperometric Biosensor Based on Laccase Immobilized in Polymer Matrices for Determining Phenolic Compounds," *Journal of Analytical Chemistry,* vol. 60, no. 6, pp. 553-557, 2005/06/01 2005, doi: 10.1007/s10809-005-0138-2.

[11] X.-H. Zhou, L.-H. Liu, X. Bai, and H.-C. Shi, "A reduced graphene oxide based biosensor for high-sensitive detection of phenols in water samples," *Sensors and Actuators B: Chemical,* vol. 181, pp. 661-667, 2013/05/01/ 2013, doi: https://doi.org/10.1016/j.snb.2013.02.021.

[12] D. Li, J. Yang, J. Zhou, Q. Wei, and F. Huang, "Direct electrochemistry of laccase and a hydroquinone biosensing application employing ZnO loaded carbon nanofibers," *RSC Advances,* 10.1039/C4RA11469H vol. 4, no. 106, pp. 61831-61840, 2014, doi: 10.1039/C4RA11469H.

[13] Y. Zhang, G.-M. Zeng, L. Tang, D.-L. Huang, X.-Y. Jiang, and Y.-N. Chen, "A hydroquinone biosensor using modified core–shell magnetic nanoparticles supported on carbon paste electrode," *Biosensors and Bioelectronics,* vol. 22, no. 9, pp. 2121-2126, 2007/04/15/ 2007, doi: https://doi.org/10.1016/j.bios.2006.09.030.

[14] D. Yuan, S. Chen, F. Hu, C. Wang, and R. Yuan, "Non-enzymatic amperometric sensor of catechol and hydroquinone using Pt-Au-organosilica@chitosan composites modified electrode," *Sensors and Actuators B: Chemical,* vol. 168, pp. 193-199, 2012/06/20/ 2012, doi: https://doi.org/10.1016/j.snb.2012.03.085.

[15] Y. Song, T. Yang, X. Zhou, H. Zheng, and S.-i. Suye, "A microsensor for hydroquinone and catechol based on a poly(3,4-ethylenedioxythiophene) modified carbon fiber

electrode," *Analytical Methods,* 10.1039/C5AY02532J vol. 8, no. 4, pp. 886-892, 2016, doi: 10.1039/C5AY02532J.
[16] J. Park, J. Kim, A. Min, and M. Y. Choi, "Fabrication of nonenzymatic electrochemical sensor based on Zn@ZnO core-shell structures obtained via pulsed laser ablation for selective determination of hydroquinone," *Environmental Research,* vol. 204, p. 112340, 2022/03/01/ 2022, doi: https://doi.org/10.1016/j.envres.2021.112340.
[17] H. Qi and C. Zhang, "Simultaneous Determination of Hydroquinone and Catechol at a Glassy Carbon Electrode Modified with Multiwall Carbon Nanotubes," *Electroanalysis,* vol. 17, no. 10, pp. 832-838, 2005, doi: 10.1002/elan.200403150.
[18] Y. Xia, K. Wang, Y. Shi, X. Gui, C. Lv, and H. Tao, "Reduced graphene oxide cross-linked L-cysteine modified glassy carbon electrode for detection of environmental pollutant of hydroquinone," *FlatChem,* vol. 25, p. 100214, 2021/01/01/ 2021, doi: https://doi.org/10.1016/j.flatc.2020.100214.
[19] T. Chen *et al.*, "Controlled synthesis of Au@Pd core-shell nanocomposites and their application for electrochemical sensing of hydroquinone," *Talanta,* vol. 198, pp. 78-85, 2019/06/01/ 2019, doi: https://doi.org/10.1016/j.talanta.2019.01.094.
[20] D.-M. Zhao, X.-H. Zhang, L.-J. Feng, L. Jia, and S.-F. Wang, "Simultaneous determination of hydroquinone and catechol at PASA/MWNTs composite film modified glassy carbon electrode," *Colloids and Surfaces B: Biointerfaces,* vol. 74, no. 1, pp. 317-321, 2009/11/01/ 2009, doi: https://doi.org/10.1016/j.colsurfb.2009.07.044.
[21] T.-W. Chen *et al.*, "Sensitive and Low-potential Electrochemical Detection of Hydroquinone Using a Nanodiamond Modified Glassy Carbon Electrode," *International Journal of Electrochemical Science,* vol. 12, no. 9, pp. 8021-8032, 2017/09/01/ 2017, doi: https://doi.org/10.20964/2017.09.05.
[22] J. Zou, J. Ma, Y. Zhang, L. Huang, and Q. Wan, "A hydroquinone sensor based on a new nanocrystals modified electrode," *Journal of Chemical Technology & Biotechnology,* vol. 89, no. 2, pp. 259-264, 2013, doi: 10.1002/jctb.4111.
[23] L. Chen, Y. Tang, K. Wang, C. Liu, and S. Luo, "Direct electrodeposition of reduced graphene oxide on glassy carbon electrode and its electrochemical application," *Electrochemistry Communications,* vol. 13, no. 2, pp. 133-137, 2011/02/01/ 2011, doi: https://doi.org/10.1016/j.elecom.2010.11.033.
[24] T. Gan, J. Sun, K. Huang, L. Song, and Y. Li, "A graphene oxide–mesoporous MnO2 nanocomposite modified glassy carbon electrode as a novel and efficient voltammetric sensor for simultaneous determination of hydroquinone and catechol," *Sensors and Actuators B: Chemical,* vol. 177, pp. 412-418, 2013/02/01/ 2013, doi: https://doi.org/10.1016/j.snb.2012.11.033.
[25] P. Karami-Kolmoti, H. Beitollahi, and S. Modiri, "Electrochemical Sensor for Simple and Sensitive Determination of Hydroquinone in Water Samples Using Modified Glassy Carbon Electrode," *Biomedicines*, vol. 11, no. 7, doi: 10.3390/biomedicines11071869.
